# Differential retention contributes to racial/ethnic disparity in U.S. academia

**DOI:** 10.1371/journal.pone.0259710

**Published:** 2021-12-01

**Authors:** Allison K. Shaw, Chiara Accolla, Jeremy M. Chacón, Taryn L. Mueller, Maxime Vaugeois, Ya Yang, Nitin Sekar, Daniel E. Stanton

**Affiliations:** 1 Department of Ecology, Evolution and Behavior, University of Minnesota-Twin Cities, Saint Paul, MN, United States of America; 2 Department of Plant and Microbial Biology, University of Minnesota-Twin Cities, Saint Paul, MN, United States of America; 3 Wildlife and Habitats Division, WWF India, New Delhi, Delhi, India; University of Utah, UNITED STATES

## Abstract

Several racial and ethnic identities are widely understood to be under-represented within academia, however, actual quantification of this under-representation is surprisingly limited. Challenges include data availability, demographic inertia and identifying comparison points. We use de-aggregated data from the U.S. National Science Foundation to construct a null model of ethnic and racial representation in one of the world’s largest academic communities. Making comparisons between our model and actual representation in academia allows us to measure the effects of retention (while controlling for recruitment) at different academic stages. We find that, regardless of recruitment, failed retention contributes to mis-representation across academia and that the stages responsible for the largest disparities differ by race and ethnicity: for Black and Hispanic scholars this occurs at the transition from graduate student to postdoctoral researcher whereas for Native American/Alaskan Native and Native Hawaiian/Pacific Islander scholars this occurs at transitions to and within faculty stages. Even for Asian and Asian-Americans, often perceived as well represented, circumstances are complex and depend on choice of baseline. Our findings demonstrate that while recruitment continues to be important, retention is also a pervasive barrier to proportional representation. Therefore, strategies to reduce mis-representation in academia must address retention. Although our model does not directly suggest specific strategies, our framework could be used to project how representation in academia might change in the long-term under different scenarios.

## Introduction

Large segments of society are under-represented in academic Science and Engineering (S&E) [1, 2]. For example, in 2017, 12% and 0.7% of the general U.S. population were Black and American Indian/Alaskan Native respectively, compared to 10% and 0.5% of students graduating with a bachelor’s degree, and 4% and 0.2% of tenured faculty [3]. Critically, the groups that are most under-represented in S&E are the fastest growing in the U.S. population [2].

Understanding and addressing mis-representation (representation that differs from a baseline expectation of proportional representation) within academia is important for numerous reasons. First, mis-representation of groups can indicate that access is not equitably distributed and that some groups have been excluded from academia [4, 5]. Second, mis-representation can mean that some of the best minds are excluded from academia [4]. Furthermore, because members of under-represented groups across various axes (gender, race, experience) can produce innovative work at higher rates than those of well represented groups [6], current mis-representation may be lowering overall academic productivity. Third, although researchers individually have unique perspectives (and thus biases), diversity across researchers can minimize collective bias and improve objectivity [4]. Finally, representation in academia can facilitate a virtuous cycle: academics, as instructors and thought-leaders, are often role-models to those considering professional scholarship, so a diverse academic environment can help draw talent from all segments of society/backgrounds [7].

A critical step in addressing mis-representation is determining where and when disparities occur. Historically, U.S. academia has been primarily composed of White scholars with under-represented minorities systematically excluded from the late 1800s through to the 1970s [2]. Although U.S. academia (especially at the undergraduate stage) has become more diverse in the past 40 years, most racial/ethnic groups are still under-represented compared to the general U.S. population [2]. Mis-representation at any stage in academia can be driven by recruitment into—as well as retention within—that stage [8, 9]. Past efforts to increase under-represented groups have primarily focused on recruitment into the undergraduate stage, and have seen limited success [10]. Increasingly there is a call for addressing factors that shape retention of under-represented groups in academia post-undergrad [9–12].

Despite its widespread existence and importance, mis-representation across academia is challenging to study for a number of reasons. First, defining an appropriate baseline for racial/ethnic minorities can be challenging. Critically, U.S. demographics are continuously changing [13], and yet academic training is a multi-decade process, which means comparisons of current academia to current census data are ignoring a potential lag effect. This heterogeneity obscures any clear targets for what diversity ‘should’ look like. Second, data are often lacking, either on the number of individuals (e.g., low sample size of under-represented groups) or over time (e.g., long enough data to look for temporal trends). Thus, many studies that aim to test for race/ethnicity-based differences often lack the sample size or statistical power [14]. Finally, analyses can only be as disaggregated as the categories underlying the data. Studies often lump together several minority groups into a broad ‘under-represented minority’ (URM) category [15].

Here, we combine two approaches to overcome these hurdles and quantify mis-representation across racial/ethnic groups and across academia. We leverage large national datasets collected by the United States National Science Foundation (NSF) on the racial and ethnic composition of all U.S. Science and Engineering academics from undergraduate students to tenured professors, spanning 25 years for students and faculty (seven years for postdocs). We generate a baseline expectation for the racial/ethnic composition of academia by developing a null model [16, 17] that dynamically accounts for historical changes in racial/ethnic compositions. Using these two tools, first, we quantify what racial/ethnic composition we would expect to see in academia, in a scenario where individuals of each race/ethnicity were equally likely to have an academic career (the null model). Second, we determine to what degree the actual representation of each racial/ethnic group in each stage of academia (e.g., doctoral student, professor) is higher, equal, or lower than that predicted by the null model. This approach allows us to control for recruitment and measure the effects of differential retention. Finally, we show that the deviance from the null model differs by racial/ethnic group and by academic stage. Our results provide a novel perspective on the status of diversity in academia, the critical role of retention, and the challenges academics continue to face.

## Methods

We constructed a model of academia in the United States as a series of stages with inputs (from the previous stage) and outputs (to the next stage or move out of academia) [18]. We parameterized our model structure with data collected by NSF for Science and Engineering fields (Biological and agricultural sciences; Earth, atmospheric, and ocean sciences; Mathematics/computer sciences; Physical sciences; Psychology; Social sciences; Engineering) for the years 1991–2016. We used our model to generate simulated ‘predictions’ of the representation we would expect of each federally categorized racial/ethnic group (Asian, Black/African-American, Native Hawaiian/Pacific Islander, Hispanic/Latino, American Indian/Alaskan Native, White, More Than One Race) in each stage of academia under the null assumption of no race/ethnicity-based differences in retention. With our approach, we can control for recruitment at one stage of academia and measure the effects of retention to future stages. That is, what ‘should’ representation in academia look like if there were no race- or ethnicity-based differences in tendency to move between stages or out of academia, and how does actual representation differ?

### Data

We used data compiled by the National Science Foundation (NSF) on the structure of academia (number of scholars in each academic stage, time spent in each stage), the racial/ethnic composition of scholars at each stage, and the approximate age distribution of scholars in each stage (see S1 File, S1–S5 Figs, S1 and S2 Tables). Data on the number of bachelors and PhD degrees came from the NSF reports on Science and Engineering Degrees [19] and Women, Minorities, and Persons with Disabilities (WMPD) [3], data on the number of graduate students and postdoctoral scholars came from the NSF Survey of Graduate Students and Postdoctorates in Science and Engineering [20], and data on the number of assistant and tenured professors came from the 2019 NSF report on Science and Engineering Indicators [21]. The length of time in each stage came from the 2018 NSF report on Science and Engineering Indicators [22] for graduate students, the NSF report on Postdoc Participation of Science, Engineering, and Health Doctorate Recipients [23] for postdocs and the integrated data system Scientists and Engineers Statistical Data System (SESTAT) for faculty.

Data on the racial/ethnic composition of undergraduate and PhD students as well as assistant and tenured professors came from the WMPD reports [3]. Data on postdoctoral researchers (2010 onward) came from NSF Surveys of Graduate Students and Postdoctorates in Science and Engineering [24], and data prior to 2010 were estimated as the average of representation in the graduate student and assistant professor stages. The student data in the NSF WMPD reports only include racial/ethnicity data for U.S. citizens and permanent residents. To account for international students, we used the NSF reports on Doctorate Recipients from U.S. Universities [24] for data on the proportion of permanent vs temporary resident PhD recipients and the racial/ethnic composition of temporary resident PhD recipients. Count data on the number of scholars of each racial/ethnic group were converted to proportions and data were smoothed with a 5-year window moving average.

Finally, we used NSF data on the approximate age range of scholars at each stage by pulling data from the SESTAT database and determining the most representative ages of each stage. These age ranges were: 15 to 24 years old (undergraduate students), 20 to 29 (graduate students), 25 to 39 (Ph.D. recipients), 25 to 44 (postdoctoral researchers), 30 to 49 (assistant professors) and 35 to 59 (tenured professors). We used these data to determine which subset of the general population we should compare each academic stage to. We determined the racial composition of the age class corresponding to each academic stage based on data from the National Center for Health Statistics and the U. S. Census Bureau [13].

### Model structure

**Fig 1:**
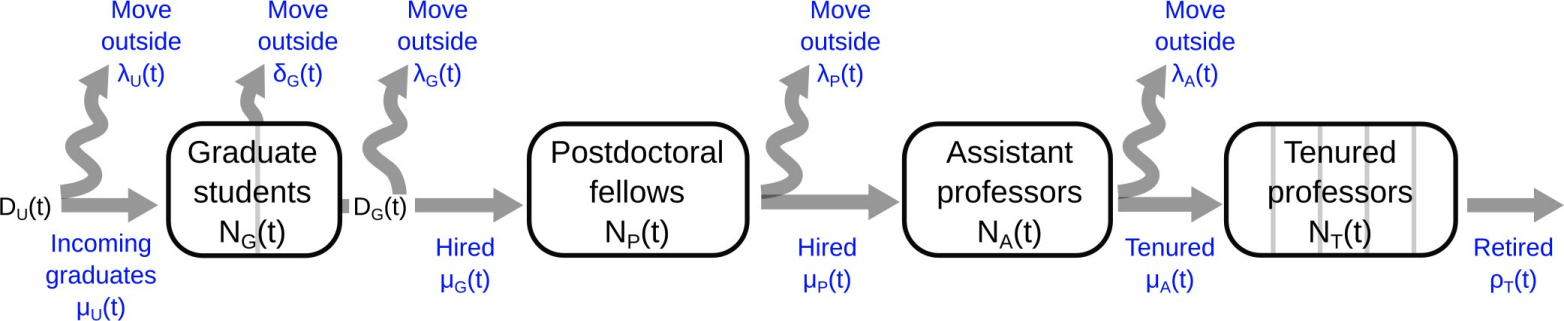
Model schematic. Academia is represented as a series of stages, where individuals either move to the next stage, or move outside of the system (academia) to other career paths. Black text indicates NSF data, blue text indicates estimated data. The stages for graduate students (G) and tenured professors (T) are split into sub-partitions (grey lines), representing pre- and post-exam stages for graduate students and equally spaced intervals for tenured professors.

We constructed a model of academia as a series of stages (Fig 1), building on previously developed methods [16]. We considered five academic stages: undergraduate students, graduate students, postdoctoral researchers, assistant professors and tenured professors. We used the time spent in each stage to estimate a turnover rate for that stage, and thus estimate the number of scholars leaving each stage in each year. Scholars that move out of each stage either move up and fill empty positions in the next stage, or move out of the system. The number of empty positions in the next stage was estimated based on the turnover rate for that stage combined with any change in the number of scholars in that stage from one year to the next (see S2 File, S6 Fig, S3 Table).

### Model simulation

We simulated the flow of scholars through our null model of academia over time, assuming there was no racial/ethnic bias in movement patterns of scholars (see S3 File, S7–S9 Figs, S3 and S4 Tables). In other words, individuals entering a given stage were drawn from the stage below in proportion to their representation in the lower stage. We initialized model simulations in a given starting year *t*_0_ with NSF data on the racial/ethnic composition of each stage in that same year. For each year going forward, we fed in NSF data on racial/ethnic composition at a particular stage (e.g., undergraduate students), and used our model to predict the racial/ethnic composition at the other stages (e.g., graduate students).

We simulated the model under four scenarios (based on turnover rate and turnover type) to capture uncertainty in the details of transitions for faculty. For turnover rate, we considered ‘slow’ (8 years spent as an assistant professor and 30 years as a tenured professor) and ‘fast’ (5 years as assistant and 20 years as tenured) turnover rates. For turnover types, we considered ‘supply’ (assistant professors achieved tenure at a specified rate, and excess tenured professors were retired accordingly), and ‘demand’ (tenured professors retired at a specified rate and excess assistant professors becoming tenured left academia) scenarios.

We ran three sets of simulations, each run under the four scenarios described. First, to consider the overall effects of retention, we initialized the model by setting the number of scholars in each class to the data from 1991 (*t*_0_ = 1991), and fed in racial/ethnic data at the undergraduate stage (and at the PhD stage for international students, the earliest stage this data were available; see above) each year until 2016, and measuring simulated output at all other stages. Second, to consider the effects of retention within each stage of academia, we again initialized the model with 1991 data, but fed in racial/ethnic data at each stage and measured the model output at the next stage (e.g, fed in graduate student data, measured postdoc data), and then took the average output across each of the four scenarios. Third, to examine how the effect of specific transitions within academia changed over time, we started simulations in different starting years and ran each simulation for 10 years. Here again we simulated expected results for each stage based on our model run with NSF data at the previous stage.

### Testing model predictions

To test our null hypothesis that there is no racial/ethnic bias in transitions within academia, we compared the racial/ethnic composition predicted by our null model to the actual composition from NSF data. To quantify relative representation, we used metric

$$\theta_{i}(t,k)=\frac{{\hat{f}}_{i}(t,k)-f_{i}(t,k)}{{\hat{f}}_{i}(t,k)}$$ [1]

where ${\hat{f}}_{i}(t,k)$ and *f*_*i*_(*t*,*k*) are the observed and simulated (respectively) fraction of individuals in stage *i* at time *t* from racial/ethnic group *k* (see S4 File). To measure confidence in our results, we considered a 5% increase or decrease in each ${\hat{f}}_{i}(t,k)$ and *f*_*i*_(*t*,*k*) values, recalculated *θ*_*i*_(*t*,*k*) for these, and mark this range of values with confidence intervals.

## Results

**Fig 2:**
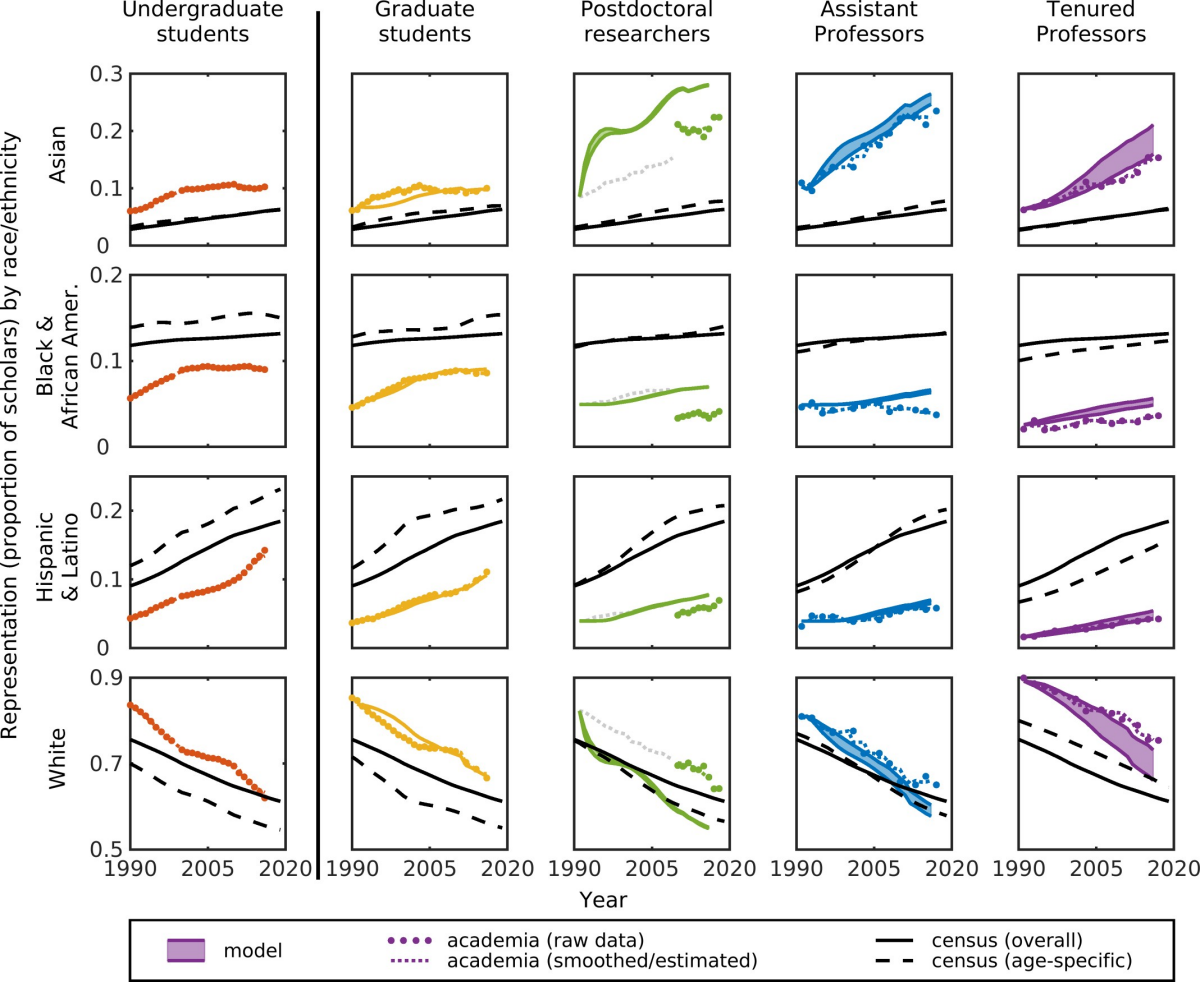
Scholar representation over time. The representation (i.e., the proportion of scholars in that stage that identify as that race or ethnicity) of the four largest American race/ethnicity categories (rows) in each academic stage (columns) over time comparing: null model predictions (colored solid lines), academia data (dots are raw data, dotted lines are smoothed data), and census data for the U.S. overall population (black solid lines) and US age-specific population (black dashed line). Mismatch between model and academia data indicate race/ethnicity-based biases of retention within academia, mismatch between model and census indicates race/ethnicity-based biases in recruitment into academia. Postdoc data before 2010 were unavailable, were estimated as the average of the graduate student and assistant professor data, and are greyed out in the figure. (See S7 Fig for additional race/ethnicity categories).

**Fig 3:**
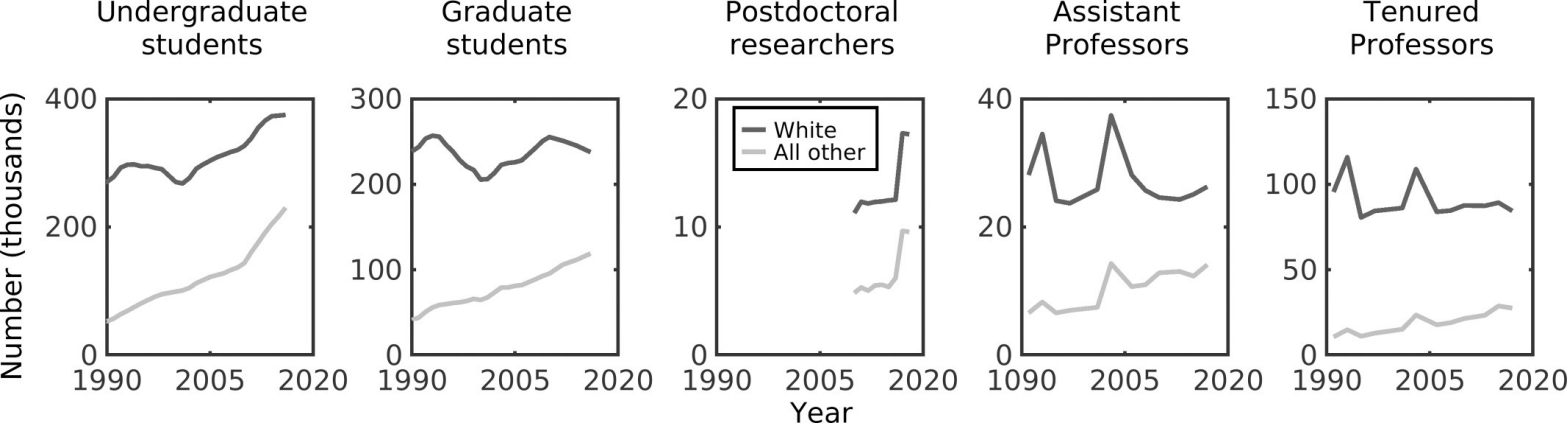
Number of scholars over time. The absolute number (in thousands) of scholars that are White (solid black line) and all other races/ethnicities (solid grey line) in each stage (panel) over time.

First, we considered the effects of retention across a full academic career while controlling for recruitment at the undergraduate stage (Fig 2). Our null model predicts that representation of scholars in most groups is still changing over time, indicating that parity would not yet have been reached, even under a null model (Fig 2 and S7 Fig, solid colored lines). We also find that increasing representation of non-White scholars (driven by changing undergraduate demographics) does not come with a decrease in the absolute number of White scholars; rather this is driven by an overall increase in the absolute number of scholars in each stage (Fig 3). Our null model predicts that representation of White scholars would be lower than levels actually observed in academia while all other groups (including Asian scholars, who are not traditionally considered an under-represented minority [3]) would be higher than observed (Fig 2, colored lines versus dots). These deviations indicate that race/ethnicity-based biases occur after graduating with a science or engineering undergraduate degree, suggesting differential retention within academia.

Second, we can compare our model results to census data, allowing us to consider the effects of recruitment, although indirectly. Here, we compare model predictions (which assume recruitment at the undergraduate stage and control for retention at other stages) with the U.S. general population census data (which included individuals who both were and were not ‘recruited’ into academia). Our null model predicts that, even if retention were the same across racial groups, representation of White and Asian scholars in academia would still be higher than in the U.S. general population while all other groups would still be lower (Fig 2 and S7 Fig, black lines). The differences between the racial composition of the null model and the general population indicate differential recruitment into academia, showing that there are race/ethnicity-based biases in entering academia. Intriguingly, taken together, our results indicate that Asian scholars can be considered over-represented in U.S. academia if the baseline for comparison is the U.S. general population, but can be considered under-represented in U.S. academia if the baseline for comparison is student degree recipients. This result is driven by the fact that many U.S. PhD recipients are international students (temporary residents; S5A and S5B Fig), and that 60+% of these students are Asian scholars (S5D Fig).

**Fig 4:**
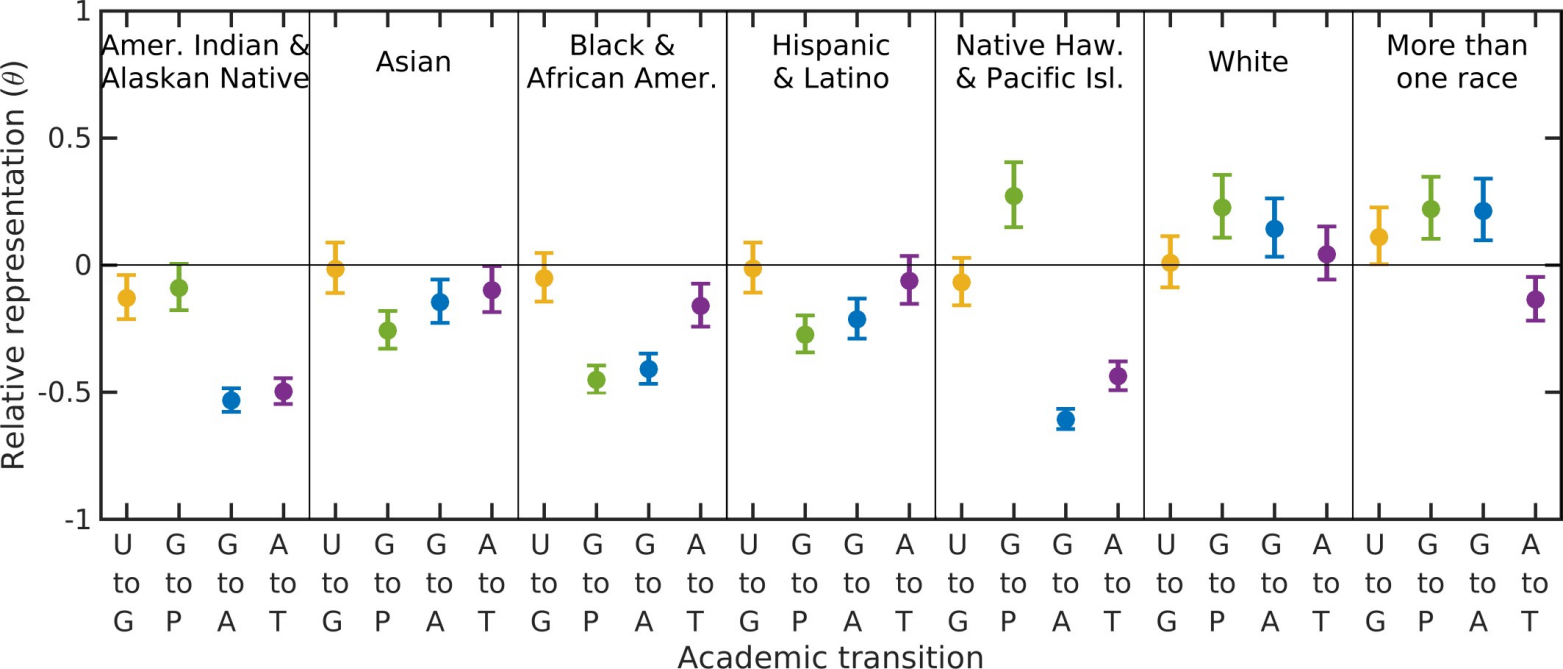
Relative representation across transitions. The relative representation (θ [Eq 1]; comparing data and the null model) over 15 years (1991–2016) of each race/ethnicity category through one of the transitions within academia: undergraduate to graduate student (U to G), graduate student to postdoctoral researcher (G to P) or to assistant professor (G to A), and assistant to tenured professor (A to T). Positive or negative values indicate a race/ethnicity category faces correspondingly positive or negative bias across that transition. Confidence intervals mark the range of θ values that result from a 5% increase or decrease in representation in either the data or model.

Third, we considered the effects of retention within each stage of academia (Fig 4). Here, we control for recruitment at each stage of academia and measure the effects of retention to each subsequent stage. We quantify relative representation (driven by retention) as a metric *θ*, the deviance from the null model in representation, where positive values (*θ* > 0) indicate a group has higher representation than the model predicts and negative values (*θ* < 0) indicate a group has lower representation than predicted. We find that *θ* varies by racial/ethnic group and by stage transition within academia (Fig 4). The transition from undergraduate degree to graduate student is captured well by the null model (*θ ≈* 0, i.e., little differential retention at this transition). The biggest loss in representation (lowest retention) for Native American/Alaskan Native and Hawaiian/Pacific Islander scholars occurs in the transition to being a faculty member and staying within the faculty (Fig 4). In contrast, the biggest loss in representation for Asian, Black and Hispanic scholars occurs in the transition from graduate student to postdoctoral researcher, and is the worst for Black representation (Fig 4). Although temporal trends over 15 years show the system is approaching parity (*θ →* 0) for some race/ethnicity and stage combinations, deviance from parity is actually increasing for Black, Hawaiian and Native scholars in faculty positions (S8 Fig).

## Discussion

The novelty of our work is three-fold: we provide new findings on the patterns, causes, and consequences of mis-representation of racial/ethnic groups within US Science and Engineering academia. In terms of patterns, we present one of the most extensive assessments of mis-representation, by contrasting one of the world’s largest public datasets on demographics of scholars with a null model of representation. Past studies have demonstrated that some racial/ethnic groups are misrepresented at some stages [15, 25], or within some disciplines [17], however it was not previously clear to what extent these observations scaled up to affect cross-discipline patterns at the national level. Here, we quantitatively show that they do. The breadth and resolution of our analysis allows us to separate effects by racial/ethnic group (rather than lumping all non-White scholars together, as past studies have done), thus demonstrating that retention at each academic stage differs by race/ethnicity. The representation patterns that we uncover also highlight the importance of explicitly defining a baseline against which to measure representation. For example, we find that although Asian representation in academia is higher than in the general U.S. population, it is simultaneously lower than would be predicted based on student demographics. Much of the racial/ethnic diversity in PhD recipients derives from immigration rather than retention of minority scholars (S5E and S5F Fig). Most international PhD students are from India and China [26] and 70% of foreign-born PhD doctorates stay in the U.S. after receiving their degree [27], which fit with our finding that non-U.S. born Asian scholars are a critical input into U.S. academia (S9 Fig).

In terms of causes, we demonstrate definitively that failed retention of Black, Indigenous, and Hispanic scholars is a substantial contributor to mis-representation in academia. We find that although representation of non-White scholars in academia is increasing, it is doing so slower than expected under our null model predictions. In other words, although recruitment into academia at the undergraduate stage is numerically the largest driver of representation, it alone does not explain the lack of parity. Our findings show that training diverse students is not enough; there is a substantial drop in racial/ethnic representation between students (graduate and undergraduate) and researchers (postdocs and faculty), and bias in retention appears to be increasing in some cases (S8 Fig, transitions to faculty for Black and Native scholars and within faculty for Native scholars). Overall, these results provide quantitative evidence to support calls for increased focus on inclusion/retention along with recruitment [9–12] and show that neither time, nor simple pushes to increase recruitment are panaceas to this societal challenge.

The patterns and causes discussed above have a number of consequences. First, failed retention in academia is most problematic for representation of Black and Indigenous scholars (Fig 4); thus, paths forward must draw on understanding the specific cultural context of these scholars as well as the challenges and discrimination that they face within academia [28–30]. Second, our finding that the most problematic transitions within academia vary by race and ethnicity indicates that different racial/ethnic groups need support at different stages [8]. Thus, policy change to address mis-representation within academia must account for the interactive effects between race/ethnicity and academic stage; a one-size-fits all solution is insufficient. Finally, it is clear that faculty do not reflect the diversity of undergraduate students., limiting the number of students who can ‘see themselves’ represented among their instructors [7].

There are three key future directions that could build on our study. First, future work could use different null models to test factors acting prior to undergraduate degrees (K-12 education, or within the undergraduate years), or to consider variants on the career trajectory we considered (e.g., removing the postdoctoral stage, allowing for time spent in industry jobs between academic positions, or more explicitly modeling variation in the time spent in different stages). Second, our approach would be greatly complemented by the collection and analysis of longitudinal datasets (tracking the same individuals over time) [Personal correspondence with Karen Hamrick (NSF-National Center for Science and Engineering Statistics) on January 5, 2021, indicating that longitudinal versions of the NSCG and SDR data are in development and are planned for future release.]. For example, as definitions of race/ethnicity change over time, scholars may move between race/ethnicity categories [31]. Non-U.S. born scholars similarly change categories: they are not counted by race/ethnicity while they are temporary residents (e.g. as students; [32]), but ‘become’ minorities with permanent residency. Longitudinal data would also help distinguish between the possible scenarios of high input and low retention versus low input and high retention. Third, future work could explore our research questions at different scales. One could ask whether representation of scholars by race/ethnicity varies across fields within S&E as is true for gender [16]. For example, Asian scholars are under-represented in Ecology even as they appear over-represented in S&E [33]. The category ‘Asian’ is incredibly broad, masking a huge amount of diversity itself [34]; different scholars having very different experiences based on cultural background and history [35]. Adopting an intersectional perspective will almost certainly change our understanding of representation [36], with many axes of identity (e.g. economic background, religion, disability, sexual orientation, gender, etc) also impacting recruitment and retention [37]. Women of color are especially likely to face distinct challenges that can be masked by considering gender and race/ethnicity separately [38]. Finally, future work could attempt to project how long it would take to reach equity in the future under varying social and policy scenarios. While this may seem like a simple extension of our model, a simplistic forecast would be misleading at best, as predicting future dynamics requires assumptions about future changes in academic labor pools and US demographics. However, our model can be adapted to provide a framework for evaluating different future scenarios and policy outcomes.

How then do we solve current mis-representation in academia? To create solutions we must draw on social, cognitive and psychological frameworks to understand the factors contributing to mis-representation [9, 39], to explicitly address the alignment of cultural identities with STEM identities [29], and to guide both intervention programs and their metrics of success [40]. It is also critical to recognize that a low relative representation in a stage can be due to problems that accumulated across earlier stages [41]; thus low representation at a particular stage may not be best served by intervention at that or the previous stage alone. Measuring whether these interventions are working will require that demographic data are collected consistently and transparently [42]. Where possible, data should be disaggregated to fully understand patterns. For example, motivational factors can vary by racial/ethnic group [28] and likely also differ with time spent in the US, especially in formative years [43] and with socio-economic and cultural background. Data collection that consistently accounts for both race/ethnicity and nativity/residence time will result in clearer understanding than current methods based on residency categories. Finally, recruitment and retention must both be addressed [44]. Recruitment into academia is not the only problem and thus a focus on increasing numbers of minority undergraduates is not enough [9]. Individuals in under-represented versus well-represented groups can have different reasons for pursuing career avenues and thus potentially different reasons for leaving academia [45].

## Conclusions

Although many academics wish to think of academia as unbiased and point to biases in earlier stages and recruitment into academia itself as driving disparities in academia [8, 10], our findings indicate this is not the case: retention within academia is critical too. Furthermore, recruiting under-represented scholars into a system (academia) that is not equipped to retain them is likely a set up for all-around failure. These findings show that neither time nor simple pushes to increase recruitment are panaceas to this societal challenge. Models identifying the impacts and extent of these biases (such as we have presented here) are a necessary part of developing and evaluating solutions. However, we should not lose sight of the fact that numbers in our model represent real people. Much work remains to address these representation problems in order to build an academia that truly reflects and realizes the potential of the society it aims to serve.

## Supporting information

S1 Fig Scholar number over time (raw). NSF time series raw data on (a) the number of bachelors degrees awarded, (b) the number of enrolled graduate students, (c) the number of PhDs awarded, (d) the number of postdoctoral researchers, (e) the number of assistant (tenure-track) professors, and (f) the number of tenured professors, across all of Science and Engineering in the US.
(TIF)

S2 Fig Scholar number over time (interpolated). Interpolated and trimmed data on (a) the number of bachelors degrees awarded, (b) the number of enrolled graduate students, (c) the number of PhDs awarded, (d) the number of postdoctoral researchers, (e) the number of assistant (tenure-track) professors, and (f) the number of tenured professors, across all of Science and Engineering in the US.
(TIF)

S3 Fig Scholar number over time (by race/ethnicity). NSF time series data on the number of (a) Asian, (b) Black, (c) Hawaiian or Pacific Islander, (d) Hispanic, (e) Native American or Alaskan Native, and (f) White individuals in each stage: undergraduate degrees, graduate students, postdoctoral researchers, assistant professors and tenured professors. Note that race/ethnicity for undergraduate and graduate students is only recorded for US citizens and permanent residents, not temporary residents (but see S5 Fig below).
(TIF)

S4 Fig Scholar representation over time (by race/ethnicity). The proportion of individuals in each stage (undergraduate degrees, graduate students, postdoctoral researchers, assistant professors and tenured professors) that are (a) Asian, (b) Black, (c) Hawaiian or Pacific Islander, (d) Hispanic, (e) Native American or Alaskan Native, and (f) White. Note that race/ethnicity for undergraduate and graduate students is only recorded for US citizens and permanent residents, not temporary residents (but see S5 Fig below).
(TIF)

S5 Fig Composition of PhD recipients by residency. The number of PhD degree awardees who are (a) U.S. citizens or permanent residents and (b) temporary residents. The fraction of each race/ethnicity among (c) U.S. citizens or permanent resident PhD recipients and (d) temporary resident PhD recipients. The fraction of scholars of race/ethnicity that are (e) U.S. citizens or permanent residents and (f) temporary residents.
(TIF)

S6 Fig Model transition estimates over time. Time series estimates of the number of individuals making each of the 10 transitions in Fig 1 of the main text, as generated by our model for each of the four transitions for faculty scenarios: ‘fast’ and ‘demand’ (blue), ‘slow’ and ‘demand’ (red), ‘fast’ and ‘supply’ (yellow), ‘slow’ and ‘supply’ (purple).
(TIF)

S7 Fig Scholar representation over time. The representation of each race/ethnicity categories (rows) in each academic stage (columns) over time (i.e. the proportion of scholars in that stage that identify as that race or ethnicity) comparing: null model predictions (colored solid lines), academia data (dots are raw data, dotted lines are smoothed data), and census data for the U.S. overall population (black solid lines) and US age-specific population (black dashed line). Mismatch between model and academia data indicate race/ethnicity-based biases of retention within academia, mismatch between model and census indicates race/ethnicity-based biases in entering academia.
(TIF)

S8 Fig Relative representation across transitions and over time. Temporal trends in the relative representation (*θ*; comparing data and the null model) of each race/ethnicity category (rows) through one of the transitions within academia (columns). Each point corresponds to a single set of simulations, which were started in one of four years *t*_1_ = 1991, *t*_2_ = 1996, *t*_3_ = 2001, *t*_4_ = 2006) and run for 10 years. Colors correspond to the stage where difference is measured (same colors as Figs 2 and 4 in the main text). Positive or negative values indicate a race/ethnicity category faces correspondingly positive or negative bias across that transition. Results for the Grad. to Postdoc. transitions are omitted for *t*_1_ and *t*_2_ because these results rely on extrapolated data, thus comparisons between model and data holds less value. Results for Hawaiian/Pacific Islander and More than one race are not shown because there were only sufficient data for a single time point (*t*_4_).
(TIF)

S9 Fig Model simulations with and without international students. The representation of each race/ethnicity category (panels) in each academic stage (lines) over time, i.e. the proportion of scholars in that stage that identify as that race or ethnicity, comparing two versions of the model. The solid lines show the main model version which accounts for the race/ethnicity of temporary resident international scholars who receive their PhDs in the U.S. (i.e., the output at the graduate student stage matches the composition of PhD recipients, regardless of their residency). The dashed lines show a version of the model that ignores the race/ethnicity of international students (i.e., the output at the graduate student stage matches the composition of U.S. citizen and permanent resident PhD recipients).
(TIF)

S1 Table Data used for our model structure, years of data, and NSF report sources. (PDF)

S2 Table Race/ethnicity data used for simulations and for comparisons against simulations, years of data, and NSF report sources. (PDF)

S3 Table Model variables, parameters, meaning and sources. (PDF)

S4 Table The fraction of individuals at each stage of each race/ethnicity in the year 2016. ‘Data’ rows are smoothed NSF counts data and the census data. The remaining rows are what the model predicts (under null model of no bias) for four scenarios: ‘fast-demand’, ‘fast-supply’, ‘slow-demand’, and ‘slow-supply’ which are combinations of a ‘demand’ or ‘supply view of faculty turnover and a `fast’ (*τ*_A_ = 5, *τ*_T_ = 20) or ‘slow’ turnover (*τ*_A_ = 8, *τ*_T_ = 30).
(PDF)

S1 File Details of data used. (PDF)

S2 File Details of model structure. (PDF)

S3 File Details of model simulations. (PDF)

S4 File Details of data reports used. (PDF)
