# Supplementary figures and images for: Differential retention contributes to racial/ethnic disparity in U.S. academia
Source: PLoS One. 2021 Dec 1;16(12):e0259710. doi: 10.1371/journal.pone.0259710 (PMC8635368; doi:10.1371/journal.pone.0259710)

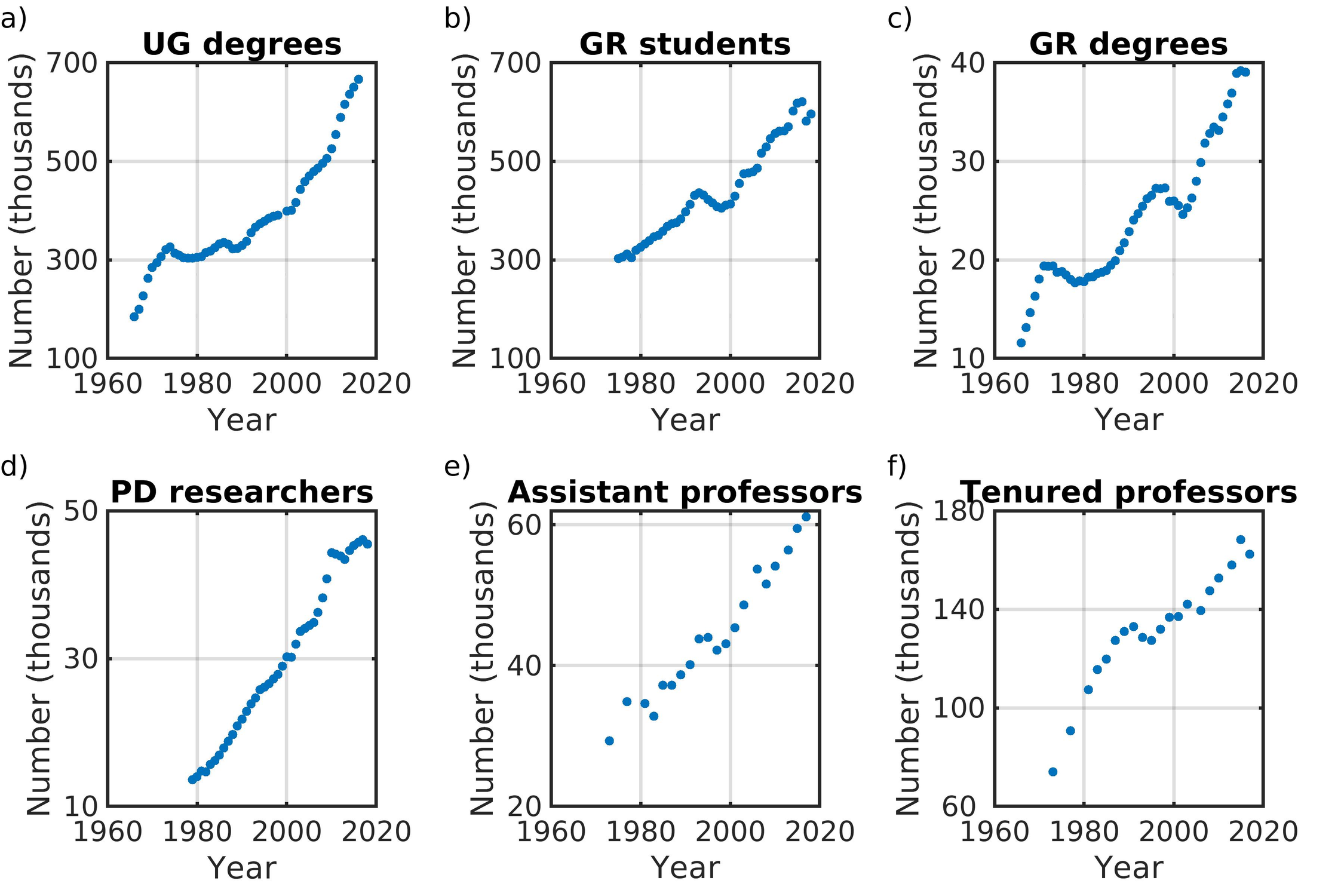

Supplement: S1 Fig — NSF time series raw data on (a) the number of bachelors degrees awarded, (b) the number of enrolled graduate students, (c) the number of PhDs awarded, (d) the number of postdoctoral researchers, (e) the number of assistant (tenure-track) professors, and (f) the number of tenured professors, across all of Science and Engineering in the US. (TIF) [file pone.0259710.s001.tif]

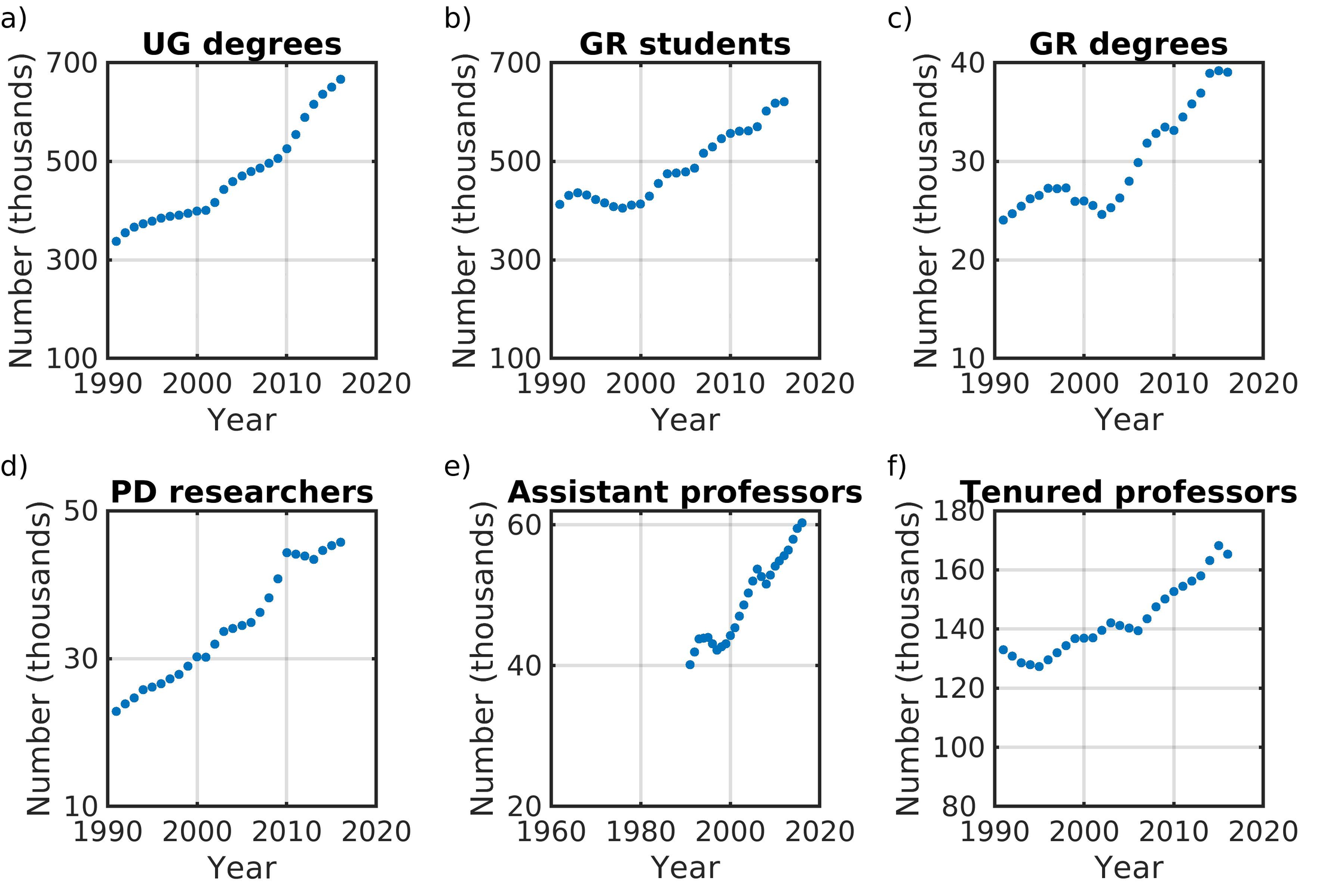

Supplement: S2 Fig — Interpolated and trimmed data on (a) the number of bachelors degrees awarded, (b) the number of enrolled graduate students, (c) the number of PhDs awarded, (d) the number of postdoctoral researchers, (e) the number of assistant (tenure-track) professors, and (f) the number of tenured professors, across all of Science and Engineering in the US. (TIF) [file pone.0259710.s002.tif]

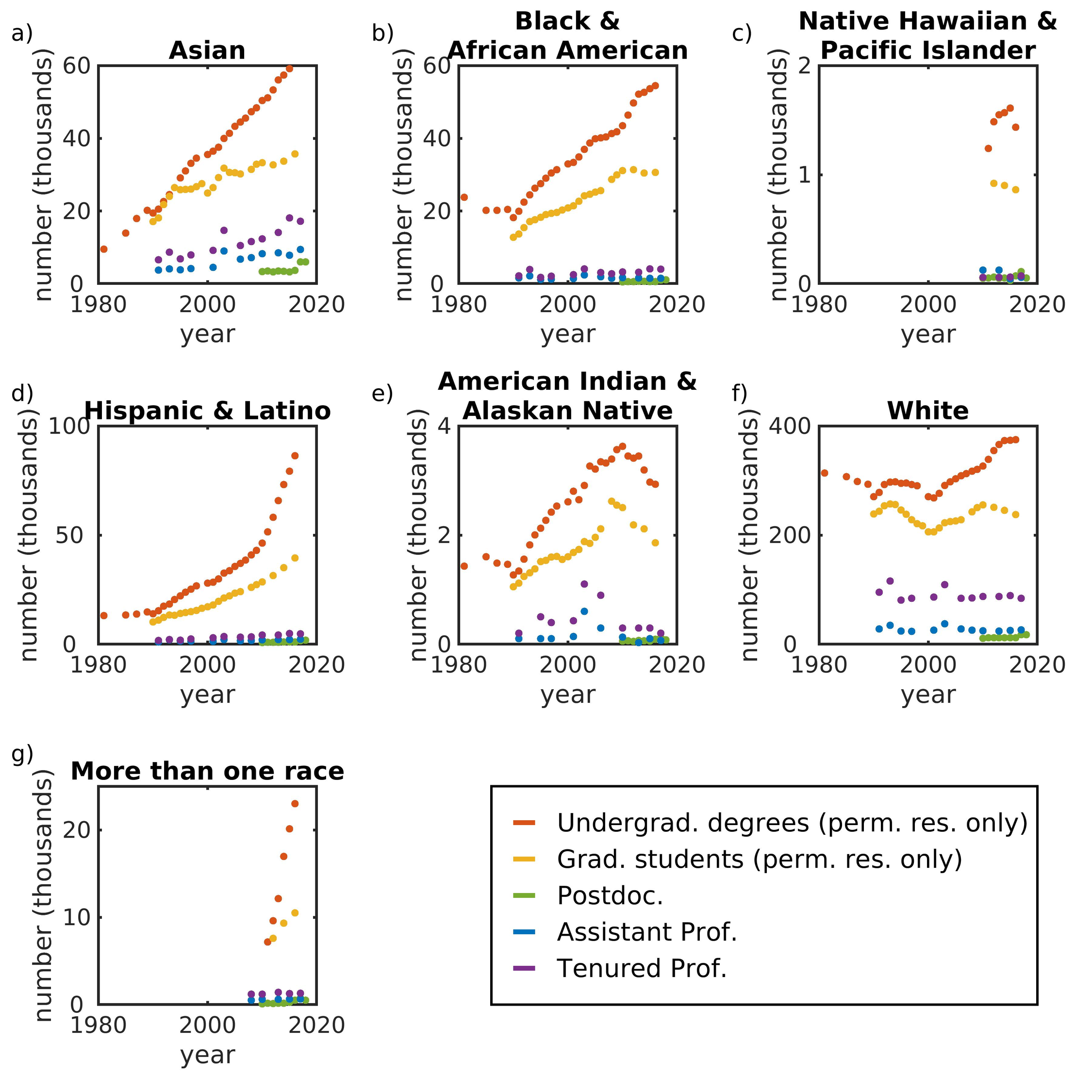

Supplement: S3 Fig — NSF time series data on the number of (a) Asian, (b) Black, (c) Hawaiian or Pacific Islander, (d) Hispanic, (e) Native American or Alaskan Native, and (f) White individuals in each stage: undergraduate degrees, graduate students, postdoctoral researchers, assistant professors and tenured professors. Note that race/ethnicity for undergraduate and graduate students is only recorded for US citizens and permanent residents, not temporary residents (but see S5 Fig below). (TIF) [file pone.0259710.s003.tif]

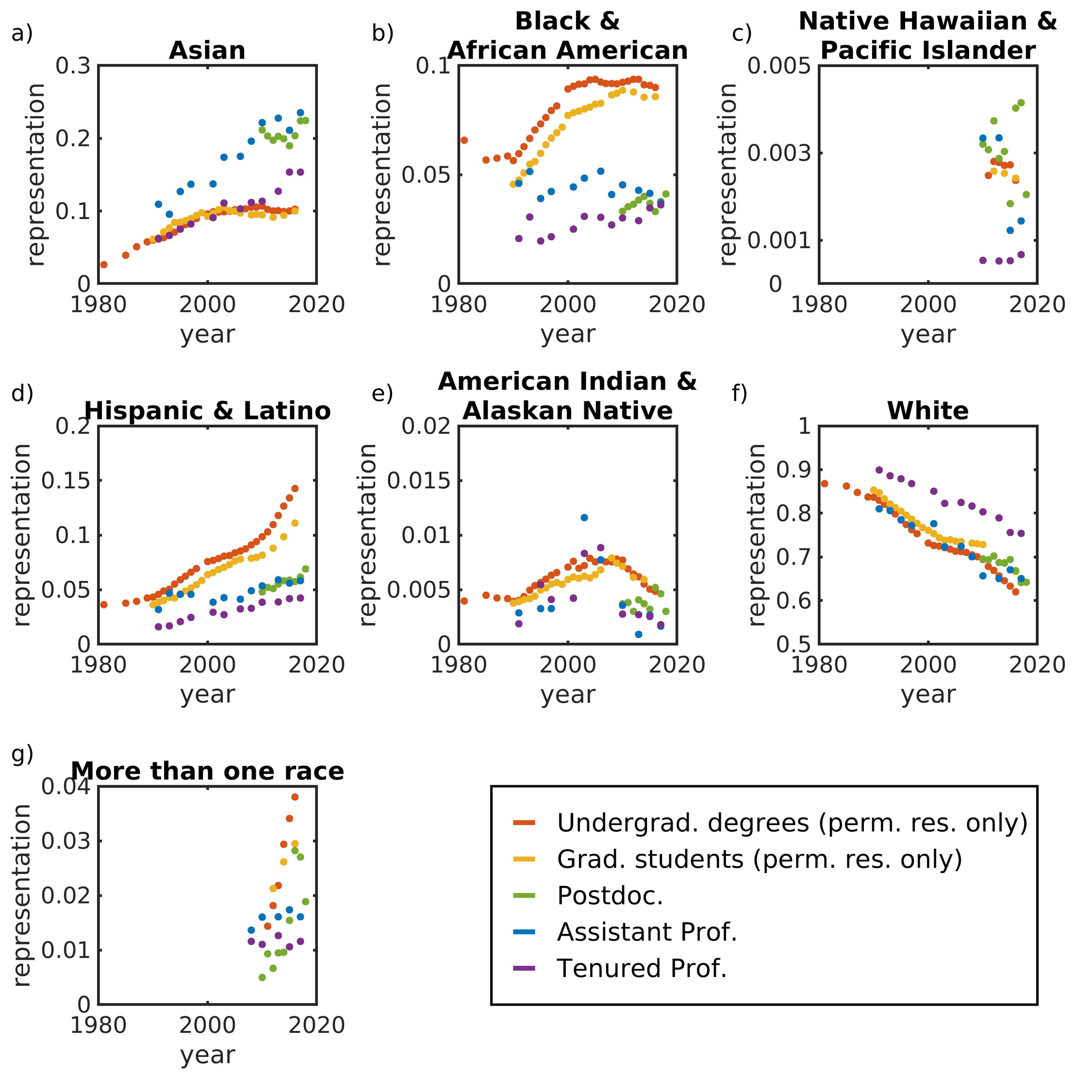

Supplement: S4 Fig — The proportion of individuals in each stage (undergraduate degrees, graduate students, postdoctoral researchers, assistant professors and tenured professors) that are (a) Asian, (b) Black, (c) Hawaiian or Pacific Islander, (d) Hispanic, (e) Native American or Alaskan Native, and (f) White. Note that race/ethnicity for undergraduate and graduate students is only recorded for US citizens and permanent residents, not temporary residents (but see S5 Fig below). (TIF) [file pone.0259710.s004.tif]

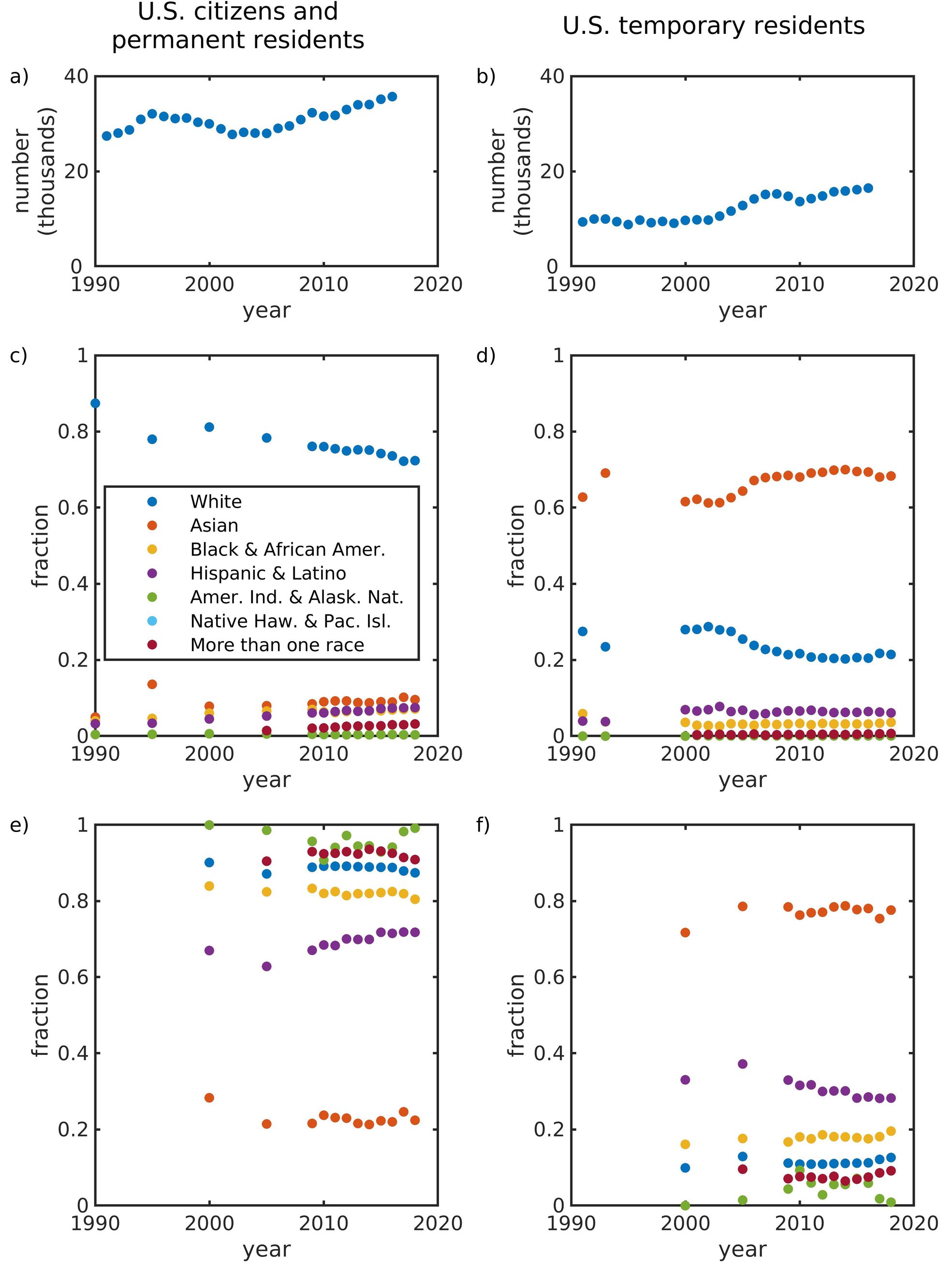

Supplement: S5 Fig — The number of PhD degree awardees who are (a) U.S. citizens or permanent residents and (b) temporary residents. The fraction of each race/ethnicity among (c) U.S. citizens or permanent resident PhD recipients and (d) temporary resident PhD recipients. The fraction of scholars of race/ethnicity that are (e) U.S. citizens or permanent residents and (f) temporary residents. (TIF) [file pone.0259710.s005.tif]

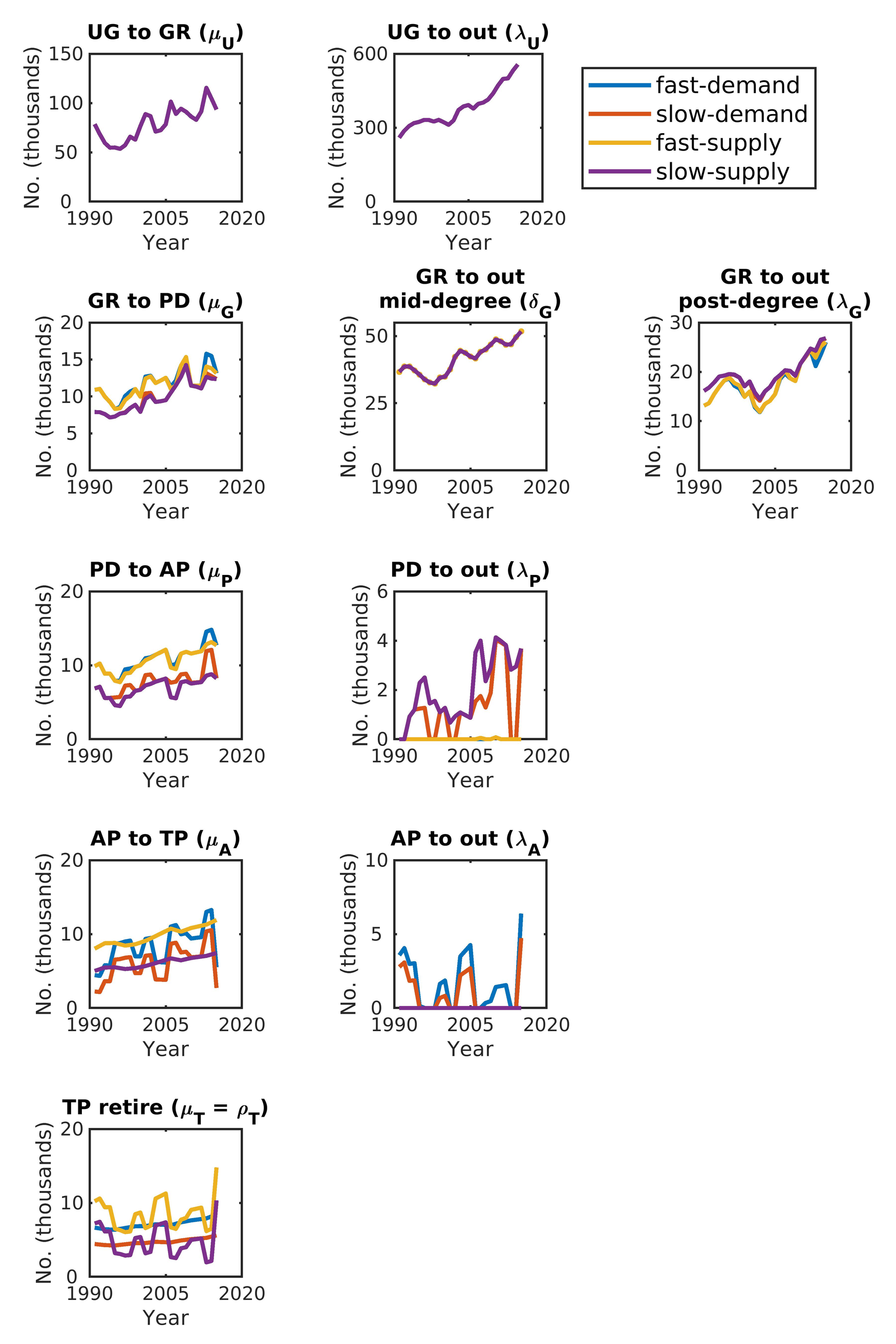

Supplement: S6 Fig — Time series estimates of the number of individuals making each of the 10 transitions in Fig 1 of the main text, as generated by our model for each of the four transitions for faculty scenarios: ‘fast’ and ‘demand’ (blue), ‘slow’ and ‘demand’ (red), ‘fast’ and ‘supply’ (yellow), ‘slow’ and ‘supply’ (purple). (TIF) [file pone.0259710.s006.tif]

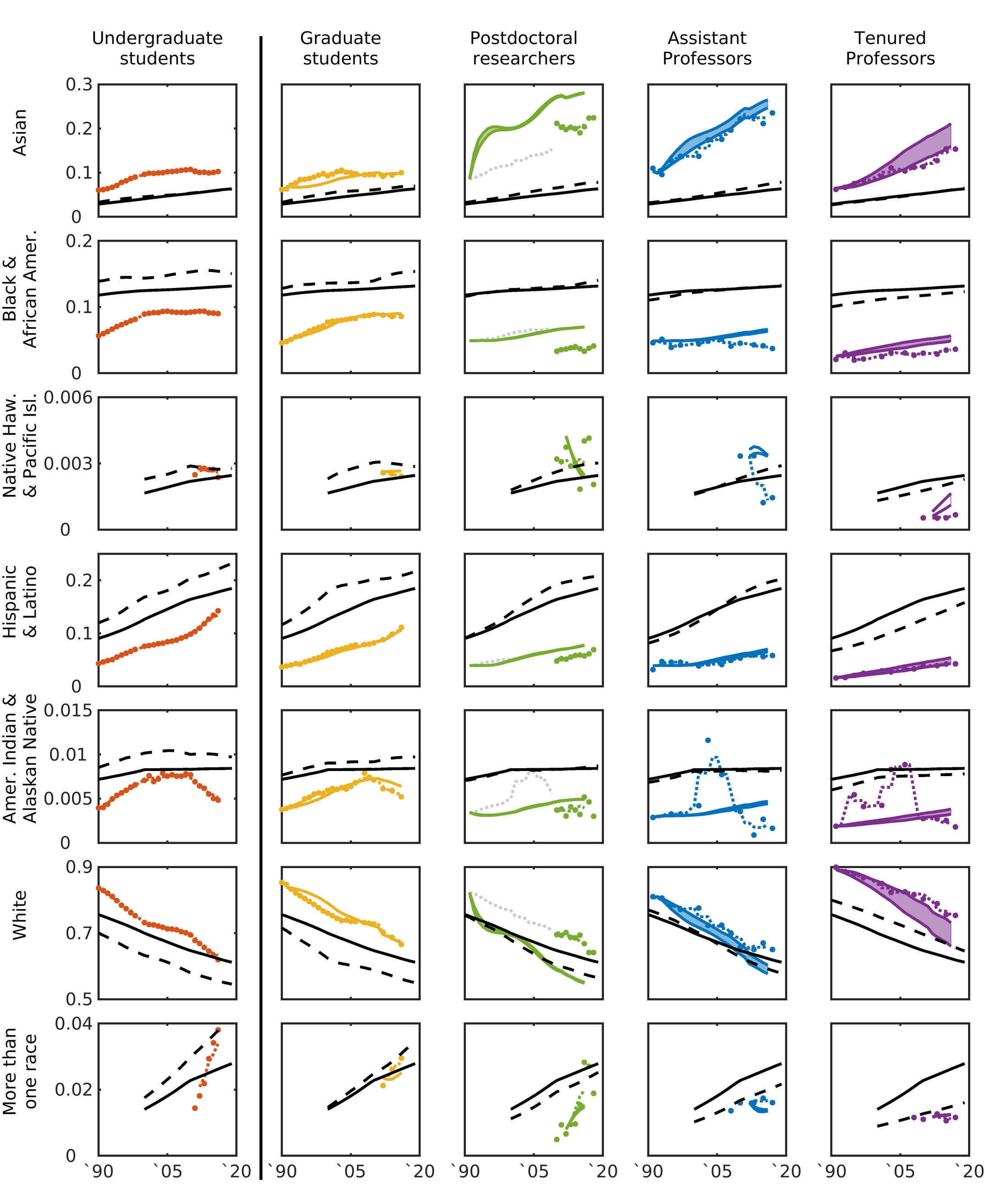

Supplement: S7 Fig — The representation of each race/ethnicity categories (rows) in each academic stage (columns) over time (i.e. the proportion of scholars in that stage that identify as that race or ethnicity) comparing: null model predictions (colored solid lines), academia data (dots are raw data, dotted lines are smoothed data), and census data for the U.S. overall population (black solid lines) and US age-specific population (black dashed line). Mismatch between model and academia data indicate race/ethnicity-based biases of retention within academia, mismatch between model and census indicates race/ethnicity-based biases in entering academia. (TIF) [file pone.0259710.s007.tif]

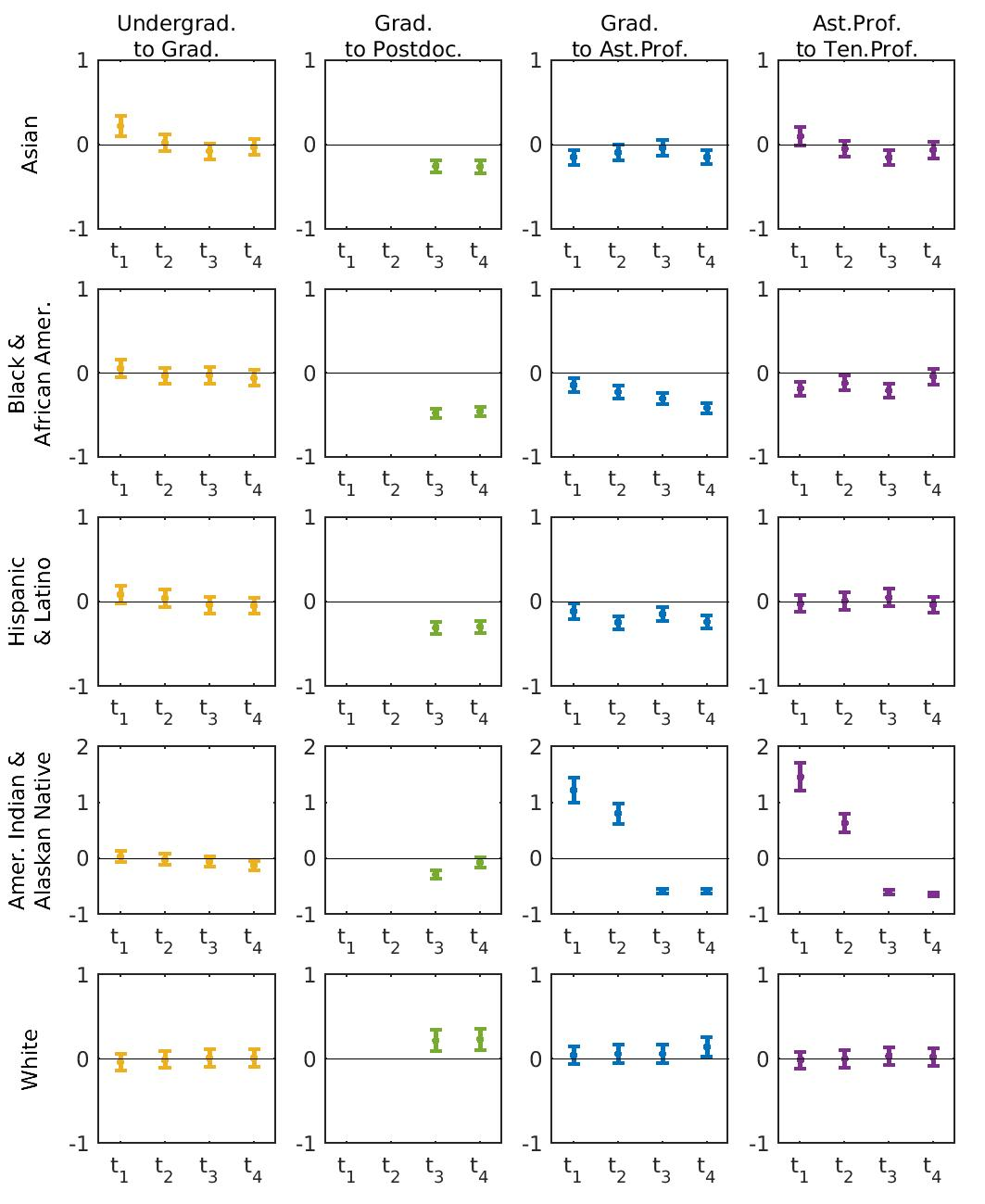

Supplement: S8 Fig — Temporal trends in the relative representation (θ; comparing data and the null model) of each race/ethnicity category (rows) through one of the transitions within academia (columns). Each point corresponds to a single set of simulations, which were started in one of four years t1 = 1991, t2 = 1996, t3 = 2001, t4 = 2006) and run for 10 years. Colors correspond to the stage where difference is measured (same colors as Figs 2 and 4 in the main text). Positive or negative values indicate a race/ethnicity category faces correspondingly positive or negative bias across that transition. Results for the Grad. to Postdoc. transitions are omitted for t1 and t2 because these results rely on extrapolated data, thus comparisons between model and data holds less value. Results for Hawaiian/Pacific Islander and More than one race are not shown because there were only sufficient data for a single time point (t4). (TIF) [file pone.0259710.s008.tif]

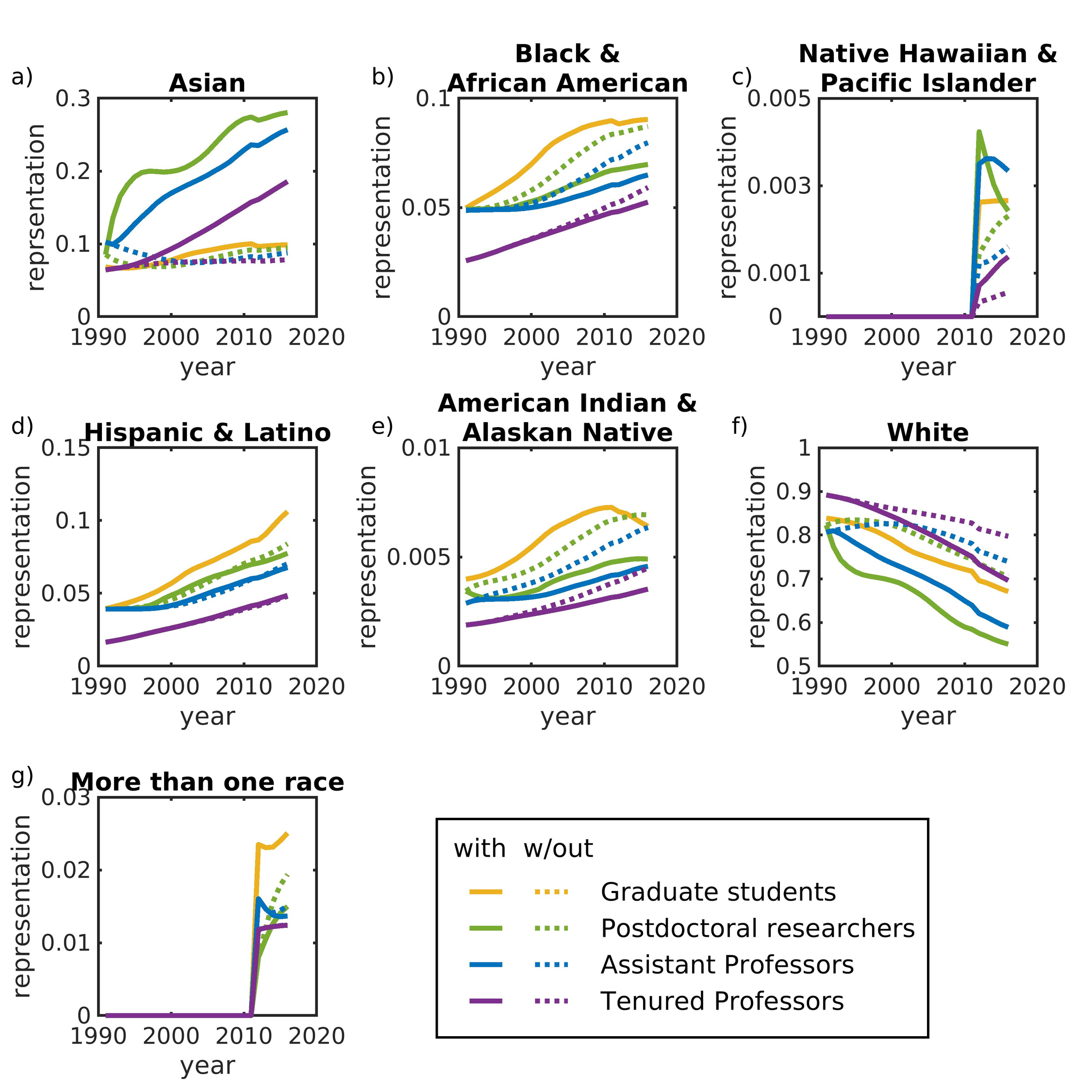

Supplement: S9 Fig — The representation of each race/ethnicity category (panels) in each academic stage (lines) over time, i.e. the proportion of scholars in that stage that identify as that race or ethnicity, comparing two versions of the model. The solid lines show the main model version which accounts for the race/ethnicity of temporary resident international scholars who receive their PhDs in the U.S. (i.e., the output at the graduate student stage matches the composition of PhD recipients, regardless of their residency). The dashed lines show a version of the model that ignores the race/ethnicity of international students (i.e., the output at the graduate student stage matches the composition of U.S. citizen and permanent resident PhD recipients). (TIF) [file pone.0259710.s009.tif]
