# Supplementary material for: Differential retention contributes to racial/ethnic disparity in U.S. academia
Source: PLoS One. 2021 Dec 1;16(12):e0259710. doi: 10.1371/journal.pone.0259710 (PMC8635368; doi:10.1371/journal.pone.0259710)
Supplement: S1 Table — (PDF) [file pone.0259710.s010.pdf]

S1 Table. Data used for our model structure, years of data, and NSF report sources.

DATA	YEARS	SOURCES
DEGREES, $D_i(t)$		
# Bachelors degrees	1966-2012<br>2006–2016	S&E Degrees, 2015 report, Table 5<br>WMPD, 2019 report, Table 7-4
# PhD degrees	1966-2012<br>2006–2016	S&E Degrees, 2015 report, Table 19<br>WMPD, 2019 report, Table 5-3
STAGE SIZE, $N_i(t)$		
# graduate students	1975-2018	GSPD, 2018 report, Tables 1-9a, 1-10a
postdoctoral researchers	1975-2018	GSPD, 2018 report, Tables 1-9b, 1-10b
assistant professors	1973-2017	SE-ind, 2019 report, Table S3-7
tenured professors	1973-2017	SE-ind, 2019 report, Table S3-7
TIME IN STAGE, τ_i		
graduate student	6.8 yrs	SE-ind, 2018 report, Table 2-30, 2015 data
postdoc	2*1.9 yrs	[08-307] 2008 report, Table 2, 2006 data
assistant professor		5-8 yrs
tenured professor		20-30 yrs
