# Supplementary material for: Differential retention contributes to racial/ethnic disparity in U.S. academia
Source: PLoS One. 2021 Dec 1;16(12):e0259710. doi: 10.1371/journal.pone.0259710 (PMC8635368; doi:10.1371/journal.pone.0259710)
Supplement: S2 Table — (PDF) [file pone.0259710.s011.pdf]

**S2 Table.** Race/ethnicity data used for simulations and for comparisons against simulations, years of data, and NSF report sources.

| DATA | YEARS | SOURCES |
|---|---|---|
| # Bachelors degrees (data by race/ethnicity for U.S. citizens and permanent residents only) | 1981-1991<br>1990-1998<br>1996-2007<br>2006-2016 | WMPD, 1994 report, Table 5-19<br>WMPD, 2002 report, Table 3-8<br>WMPD, 2009 report, Table C6<br>WMPD, 2009 report, Table 5-3 |
| # PhD students (data by race/ethnicity for U.S. citizens and permanent residents only) | 1990-1999<br>1999-2006<br>2008-2010<br>2012<br>2014<br>2016 | WMPD, 2002 report, Table 4-6<br>WMPD, 2009 report, Table D-1<br>WMPD, 2011 report, Table 3-1<br>WMPD, 2013 report, Table 3-1<br>WMPD, 2017 report, Table 3-1<br>WMPD, 2019 report, Table 3-1 |
| # PhD degrees (data by residency, permanent vs. temporary) used to calculate $R(t)$ | 1984:5:2014<br>1985:5:2015<br>1986:5:2016<br>1987:5:2017<br>1988:5:2018 | SED, 2014 report, Table 17<br>SED, 2015 report, Table 17<br>SED, 2016 report, Table 17<br>SED, 2017 report, Table 17<br>SED, 2018 report, Table 17 |
| # PhD degrees (data by race/ethnicity for temporary residents) used to calculate $V(t,k)$ | 2000–2010<br>2009–2018 | SED, 2010 report, Table 19<br>SED, 2018 report, Table 19 |
| doctoral workforce (data by race/ethnicity for temporary residents) used to calculate $V(t,k)$ | 1991<br>1993 | WMPD, 1994 report, Table 8-11<br>WMPD, 1996 report, Table 5-33 |
| # postdoctoral researchers (data by race/ethnicity) | 2010<br>2011-2016<br>2017<br>2018 | GSPD, 2010 report, Table 34<br>GSPD, 2010 report, Table 34<br>GSPD, 2017 report, Table 2-2<br>GSPD, 2018 report, Table 2-2 |
| # professors [assistant, tenured] (data by race/ethnicity) | 1991<br>1993<br>1995<br>1997<br>2001<br>2003<br>2006<br>2008<br>2010<br>2013<br>2015<br>2017 | WMPD, 1994 report, Table 8-18<br>WMPD, 1996 report, Table 5-28<br>WMPD, 1998 report, Table 5-10<br>WMPD, 2000 report, Table 5-19<br>WMPD, 2004 report, Table H-26<br>WMPD, 2007 report, Table H-28<br>WMPD, 2009 report, Table H-28<br>WMPD, 2011 report, Table 9-26<br>WMPD, 2013 report, Table 9-26<br>WMPD, 2015 report, Table 9-26<br>WMPD, 2017 report, Table 9-26<br>WMPD, 2019 report, Table 9-26 |
