# Supplementary material for: Differential retention contributes to racial/ethnic disparity in U.S. academia
Source: PLoS One. 2021 Dec 1;16(12):e0259710. doi: 10.1371/journal.pone.0259710 (PMC8635368; doi:10.1371/journal.pone.0259710)
Supplement: S3 Table — (PDF) [file pone.0259710.s012.pdf]

S3 Table. Model variables, parameters, meaning and sources.

PARAMETER	MEANING	SOURCE
t	time (year)	NA
i	stage (U,G,P,A,T)	NA
k	individual race/ethnicity	NA
$N_i(t)$	number of individuals in stage i in year t	see Table S1
$D_i(t)$	number of degrees of stage i awarded in year t (only i=U,G)	see Table S1
$R(t)$	fraction of PhD degrees to U.S. citizens / permanent residents in year t	see Table S2
$V(t,k)$	fraction of U.S. temporary resident PhD recipients in year t that are of race/ethnicity k	see Table S2
τ_i	average number of years spent in stage i	see Table S1
$\rho_i(t)$	number of individuals potentially leaving stage i in year t	estimated
$\omega_i(t)$	number of openings available in stage i in year t	estimated
$\mu_i(t)$	individuals moving from stage i to stage i+1 in year t	estimated
$\lambda_i(t)$	individuals moving from stage i to outside the system in year t	estimated
$\delta_G(t)$	individuals leaving stage G (before degree) in year t	estimated
$\beta_i(t,j)$	individuals moving from sub-partition j in stage i in year t	estimated
$n_i(t,k)$	number of k individuals in stage i in year t	simulated
$f_i(t,k)$	fraction of individuals in stage i in year t of race/ethnicity k	simulated
