# Supplementary material for: Differential retention contributes to racial/ethnic disparity in U.S. academia
Source: PLoS One. 2021 Dec 1;16(12):e0259710. doi: 10.1371/journal.pone.0259710 (PMC8635368; doi:10.1371/journal.pone.0259710)
Supplement: S4 Table — ‘Data’ rows are smoothed NSF counts data and the census data. The remaining rows are what the model predicts (under null model of no bias) for four scenarios: ‘fast-demand’, ‘fast-supply’, ‘slow-demand’, and ‘slow-supply’ which are combinations of a ‘demand’ or ‘supply view of faculty turnover and a `fast’ (τA = 5, τT = 20) or ‘slow’ turnover (τA = 8, τT = 30). (PDF) [file pone.0259710.s013.pdf]

S4 Table. The fraction of individuals at each stage of each race/ethnicity in the year 2016. 'Data' rows are smoothed NSF counts data and the census data. The remaining rows are what the model predicts (under null model of no bias) for four scenarios: 'fast-demand', 'fast-supply', 'slow-demand', and 'slow-supply' which are combinations of a 'demand' or 'supply' view of faculty turnover and a 'fast' ($\tau_A = 5$, $\tau_T = 20$) or 'slow' turnover ($\tau_A = 8$, $\tau_T = 30$).

	Asian	Black & African American	Native Hawaiian & Pacific Islander	Hispanic & Latino	American Indian & Alaskan Native	White	More than one race
U.S. general population (census)							
data	0.0602	0.1305	0.0018	0.1779	0.0084	0.6230	0.0209
Undergraduate Students							
data	0.1007	0.0906	0.0026	0.1345	0.0051	0.6327	0.03388
Graduate Students							
data	0.0973	0.0856	0.0025	0.1047	0.0056	0.6765	0.0278
fast-demand	0.0988	0.0903	0.0027	0.1062	0.0064	0.6707	0.0251
fast-supply	0.0988	0.0903	0.0027	0.1062	0.0064	0.6707	0.0251
slow-demand	0.0988	0.0903	0.0027	0.1062	0.0064	0.6707	0.0251
slow- supply	0.0988	0.0903	0.0027	0.1062	0.0064	0.6707	0.0251
Postdoctoral Researchers							
data	0.2082	0.0378	0.0030	0.0610	0.0040	0.6662	0.0198
fast-demand	0.2812	0.0698	0.0023	0.0779	0.0049	0.5486	0.0153
fast-supply	0.2807	0.0698	0.0024	0.0776	0.0049	0.5495	0.0151
slow-demand	0.2799	0.0696	0.0025	0.0772	0.0049	0.5510	0.0148
slow- supply	0.2798	0.0696	0.0025	0.0772	0.0049	0.5513	0.0148
Assistant Professors							
data	0.2230	0.0392	0.0013	0.0571	0.0022	0.6604	0.0167
fast-demand	0.2648	0.0663	0.0033	0.0700	0.0047	0.5774	0.0135
fast-supply	0.2635	0.0660	0.0034	0.0695	0.0047	0.5795	0.0135
slow-demand	0.2525	0.0641	0.0033	0.0665	0.0045	0.5953	0.0137
slow- supply	0.2468	0.0632	0.0033	0.0649	0.0044	0.6034	0.0140
Tenured Professors							
data	0.1537	0.0354	0.0006	0.0422	0.0022	0.7548	0.0111
fast-demand	0.2053	0.0553	0.0015	0.0527	0.0037	0.6690	0.0124
fast-supply	0.2110	0.0564	0.0016	0.0542	0.0038	0.6604	0.0125
slow-demand	0.1663	0.0493	0.0012	0.0441	0.0033	0.7235	0.0124
slow- supply	0.1599	0.0487	0.0011	0.0432	0.0032	0.7315	0.0123
