# Supplementary material for: Differential retention contributes to racial/ethnic disparity in U.S. academia
Source: PLoS One. 2021 Dec 1;16(12):e0259710. doi: 10.1371/journal.pone.0259710 (PMC8635368; doi:10.1371/journal.pone.0259710)
Supplement: S1 File — (PDF) [file pone.0259710.s014.pdf]

Supplementary File S1 for

Differential retention contributes to racial/ethnic disparity in U.S. academia

Allison K. Shaw[1*], Chiara Accolla[1], Jeremy M. Chacón[1], Taryn L. Mueller[1], Maxime Vaugeois[1], Ya Yang[2], Nitin Sekar[3], Daniel E. Stanton[1]

[1]Department of Ecology, Evolution and Behavior, University of Minnesota-Twin Cities, Saint Paul, MN 55108
[2]Department of Plant and Microbial Biology, University of Minnesota-Twin Cities, Saint Paul, MN 55108
[3]Wildlife and Habitats Division, WWF India, New Delhi, Delhi 110003, India *To whom correspondence should be addressed; E-mail: ashaw@umn.edu.

Data

We used three broad types of data from the National Science Foundation (NSF) in our work: (i) data on the structure of academia (number of scholars in each academic stage, time spent in each stage), (ii) data on the racial/ethnic composition of scholars at each stage, and (iii) data on the approximate age range of academics. Whenever there were multiple versions of the same data available for a given year (e.g., in different versions on the same report, or when classifications changed within a time series), we used the most recent data for a given year. We limited our analysis to the period 1991-2016 where almost all data were available (except for racial/ethnic data on postdoctoral researchers which were only available for 2010 onward).

Structural Data

The structural data we used consisted of time series of the number of bachelors and PhD degrees awarded, the number of enrolled graduate students, and the number of employed postdoctoral researchers, assistant professors and tenured professors, as well as estimates of the length of time spent as a graduate student, postdoctoral researcher, assistant professor and tenured professor. Data on the number of bachelors and PhD degrees came from the NSF reports on Science and Engineering Degrees (1), and Women, Minorities, and Persons with Disabilities (WMPD) (2), data on the number of graduate students and postdoctoral scholars came from the NSF Survey of Graduate Students and Postdoctorates in Science and Engineering (3), and data on the number of assistant and tenured professors came from the NSF report on Science and Engineering Indicators (4).

The length of time in each stage came from the NSF report on Science and Engineering Indicators (5) for graduate students, the NSF report on Postdoc Participation of Science, Engineering, and Health Doctorate Recipients (6) for postdocs and the integrated data system Scientists and Engineers Statistical Data System (SESTAT) for faculty. The specific sources for all structural data are given in Table S1 and in File S4 "Data Report Details", and the time series of structural data are plotted in Figure S1. Missing data were linearly interpolated; for example, faculty data were only collected approximately every two years, and undergraduate data were missing for the year 1999 (data with interpolation given in Figure S2).

Race/Ethnicity Data

The racial/ethnicity data we used consisted of time series data for the number of earned bachelors degrees, enrolled graduate students, and employed postdoctoral researchers, assistant professors and tenured professors by race/ethnicity. From 1991 to around 2010 NSF used five groups for race/ethnicity: 'White', 'Asian or Pacific Islander', 'Black', 'Hispanic', and 'Native American/Alaskan Native' (plus an additional group for unknown). Around 2010, the group 'Asian or Pacific Islander' was split into 'Asian' and 'Native Hawaiian or Other Pacific Islander'. At the same time, the group 'More than one race' was added. When the number of individuals in a group was quite small (this occurred for both Native American / Alaskan Native and Native Hawaiian / Pacific Islander in both assistant professor and tenured professor stages in some years) the specific number of individuals was masked instead of being reported. In these cases, we estimated the number of individuals from other group data. For example, if the total number of individuals of a race/ethnicity was reported for faculty as a whole, we split this number evenly among groups to approximate the number of individuals of that race/ethnicity in each faculty stage. Data on the racial/ethnic composition of undergraduate and PhD students as well as assistant and tenured professors came from the WMPD reports (2). Data on postdoctoral researchers (2010 onward) came from NSF Surveys of Graduate Students and Postdoctorates in Science and Engineering (3), and data prior to 2010 was estimated as the average of representation in the graduate student and assistant professor stages. The student data in the NSF WMPD reports only include racial/ethnicity data for U.S. citizens and permanent residents. To account for international students, we used the NSF reports on Doctorate Recipients from U.S. Universities (7) for data on the proportion of permanent vs temporary resident PhD recipients and the racial/ethnic composition of temporary resident PhD recipients. The specific sources for all race/ethnicity data are given in Table S2 and in the File S4 "Data Report Details", and the time series of race/ethnicity data are plotted in Figures S3, S4, and S5. Count data on the number of scholars of each racial/ethnic group were converted to proportions and data were smoothed with a 5-year window moving average.

The specific number of individuals reported for each race/ethnicity group was not necessarily representative of the actual number of individuals of that race/ethnicity, for two main reasons. First, some individuals did not report their race/ethnicity (often reported as a separate group, 'unknown'). Second, race/ethnicity data for undergraduate and graduate students were only provided for U.S. citizens and permanent residents;

race/ethnicity for temporary residents was not recorded. However, race/ethnicity data for U.S. temporary residents were recorded for graduating PhD students (see Figure S5). Thus, when applying the race/ethnicity data, we used the proportion of individuals of each race/ethnicity rather than the actual count data (plotted in Figure S4). We calculated proportions using only data for a known race/ethnicity (i.e., we excluded the 'unknown race' group). For example, if there were 500 individuals in a stage, of which 150 were White, and 50 Asian, and 300 unknown race/ethnicity, we recorded this stage as being 0.75 White and 0.25 Asian. Finally data were smoothed with the 'smoothdata' function in Matlab, using a moving average over a window of size 5 years and omitting missing data.

Age Data

Finally, we used NSF data on the approximate age range of scholars at each stage by pulling data from the integrated data system SESTAT (Scientists and Engineers Statistical Data System, https://www.nsf.gov/statistics/sestat/), and determining the most representative ages of each stage. We selected the National Survey of Recent College Graduates (NSRCG) for undergraduate and graduate stages (year 2010), and the Survey of Doctorate Recipients (SDR) for postdoc, assistant and tenured professor stages (year 2015). For undergraduate and graduate students we created a table showing the most recent degree type (labeled "M_ED_MR_DEGREE_TYPE") in function of ages ("U_DEM_AGE_RCG_PUB"), and specified the population by the field of study for the most recent degree ("M_ED_MR_MAJOR_ED_GRP_MAJOR_NEW"). We selected the fields (i) biological, agricultural and environmental life sciences, (ii) physical Sciences, (iii) computer and mathematical sciences, and (iv) engineering. The total number of scholars per age class in the undergraduate stage was calculated as the sum of Bachelor and Master degrees across the four fields. Similarly, the total number of graduate scholars was obtained by summing up the number of doctorate degrees in each field. Then, we plotted the total number of undergraduate and graduate scholars in function of age, and selected the most representative time spent in each of these two stages. We applied the same method for the other three stages. Notably, we created a table considering the academic position of postdoc ("E_JOB_EMPLR_ACAD_POSITION_POSTDOC") or tenure status ("E_JOB_EMPLR_EDUC_INST_TENURE_STAT"), in function of ages grouped by 5-year intervals ("U_DEM_AGE_GROUP_5_YR_GROUPING_PUB"), and specified the population by the field of study for the highest degree ("O_ED_HD_MAJOR_ED_GRP_MAJOR_NEW"). Overall, the age ranges we used were: 15 to 24 years old (undergraduate students), 20 to 29 (graduate students), 25 to 39 (Ph.D. recipients), 25 to 44 (postdoctoral researchers), 30 to 49 (assistant professors) and 35 to 59 (tenured professors). We used these data to determine which subset of the general population we should compare each academic stage to.

Next, we determined the racial composition of the age class corresponding to each academic stage based on data from the National Center for Health Statistics and the U. S. Census Bureau (8). To compute our racial composition by academic stage for the "American Indian/Alaska Native', "Asian", "Black/African American", "White", and "Hispanic/Latino' categories from 1990 to 2016, we compiled estimates of resident pop-

ulation of the US by year, single-year of age, bridged-race category, and Hispanic origin produced by the National Center for Health Statistics under a collaborative arrangement with the U. S. Census Bureau (8). We compiled similar data from 2000 to 2016 for the "Native Hawaiian/Pacific Islander" and "Two or More Races" categories from the 2019 Population Estimates by Age, Sex, Race and Hispanic Origin (9) and National Intercensal Tables: 2000-2010, both produced by the U. S. Census Bureau (10).

See File S2 for how each dataset was used.

References

[1] National Science Foundation, National Center for Science and Engineering Statistics. Science and Engineering Degrees: 1966–2012. Detailed Statistical Tables NSF 15-326. Arlington, VA; 2015. Available from: http://www.nsf.gov/statistics/2015/nsf15326/.

[2] National Science Foundation, National Center for Science and Engineering Statistics. Women, Minorities, and Persons with Disabilities in Science and Engineering: 2019. Special Report NSF 19-304. Alexandria, VA; 2019. Available from: https://www.nsf.gov/statistics/wmpd.

[3] National Center for Science and Engineering Statistics. Survey of Graduate Students and Postdoctorates in Science and Engineering; 2018. Available from: http://ncsesdata.nsf.gov/gradpostdoc/.

[4] National Science Board NSF. Higher Education in Science and Engineering. Science and Engineering Indicators 2020. NSB-2019-7.; 2019. NSB-2019-7. Available from: https://ncses.nsf.gov/pubs/nsb20197/.

[5] National Science Board. Science and Engineering Indicators 2018. NSB-2018-1. Alexandria, VA: National Science Foundation; 2018. Available from: https://www.nsf.gov/statistics/indicators/.

[6] National Science Foundation, Division of Science Resources Statistics. Postdoc Participation of Science, Engineering, and Health doctorate Recipients; 2008. Available from: http://www.nsf.gov/statistics/infbrief/nsf08307.

[7] National Center for Science and Engineering Statistics NSF. Doctorate Recipients from U.S. Universities: 2018. Special Report NSF 20-301. Alexandria, VA; 2019. Available from: https://ncses.nsf.gov/pubs/nsf20301/.

[8] United States Department of Health and Human Services (US DHHS), Centers for Disease Control and Prevention (CDC), National Center for Health Statistics (NCHS). Bridged-Race Population Estimates, United States July 1st resident population by state, county, age, sex, bridged-race, and Hispanic origin. Compiled from 1990-1999 bridged-race intercensal population estimates (released by NCHS

on 7/26/2004); revised bridged-race 2000-2009 intercensal population estimates (released by NCHS on 10/26/2012); and bridged-race Vintage 2019 (2010-2019) postcensal population estimates (released by NCHS on 7/9/2020). Available on CDC WONDER Online Database. Accessed at http://wonder.cdc.gov/bridged-race-v2019.html on Oct 9, 2020; 2020.

[9] Annual Estimates of the Resident Population by Sex, Age, Race, and Hispanic Origin for the United States: April 1, 2010 to July 1, 2019 (NC-EST2019-ASR6H). U.S. Census Bureau, Population Division. June 2020; 2020.

[10] Intercensal Estimates of the Resident Population by Sex, Race, and Hispanic Origin for the United States: April 1, 2000 to July 1, 2010 (US-EST00INT-02). U.S. Census Bureau, Population Division. September 2011.; 2011.
