# Supplementary material for: Differential retention contributes to racial/ethnic disparity in U.S. academia
Source: PLoS One. 2021 Dec 1;16(12):e0259710. doi: 10.1371/journal.pone.0259710 (PMC8635368; doi:10.1371/journal.pone.0259710)
Supplement: S2 File — (PDF) [file pone.0259710.s015.pdf]

Supplementary File S2 for

Differential retention contributes to racial/ethnic disparity in U.S. academia

Allison K. Shaw[1*], Chiara Accolla[1], Jeremy M. Chacón[1], Taryn L. Mueller[1], Maxime Vaugeois[1], Ya Yang[2], Nitin Sekar[3], Daniel E. Stanton[1]

[1]Department of Ecology, Evolution and Behavior, University of Minnesota-Twin Cities, Saint Paul, MN 55108
[2]Department of Plant and Microbial Biology, University of Minnesota-Twin Cities, Saint Paul, MN 55108
[3]Wildlife and Habitats Division, WWF India, New Delhi, Delhi 110003, India *To whom correspondence should be addressed; E-mail: ashaw@umn.edu.

Model structure

We constructed a model of academia as a series of stages with movement between them or out of the system (Figure 1 main text; modified from (1). Our model has five discrete stages: undergraduate studies (U), graduate studies (G), postdoctoral fellowships (P), assistant professorships (A; tenure-track) and tenured professorships (T). Individuals that move out of each stage either move up and fill empty positions in the next stage, or move out of the system.

We generated the structure of our model from NSF data. The number of academics in the U.S. has changed over time, so we set the size of each stage i in each year t ($N_i(t)$) from data on the actual number graduate students, postdoctoral fellows, assistant professors, and tenured professors, using data from NSF reports (see Table S1 for specific data sources, and Figure S2 for data). We used the time spent in each stage to estimate a turnover rate for that stage which, in combination with the number of scholars in each stage, gave us an estimate of the number of scholars that would have either transitioned from one stage to the next or transitioned outside of the system for each year.

Estimating Transitions

Each of the transitions was estimated as follows (see Figure S6 for results). For each year and each stage, we estimated the number of individuals leaving each stage based on

transition rates and changes in stage sizes as

$$\rho_i(t) = \left(\frac{1}{\tau_i}\right) N_i(t) \tag{S1}$$

for stages $i = \{\text{G, P, A, T}\}$ where τ_i is the average number of years spent in stage i (see Table S3 for all model parameters). Simultaneously, we estimated the number of openings in each year and each stage as

$$\omega_i(t) = N_i(t+1) - N_i(t) + \rho_i(t) \ . \tag{S2}$$

In most cases, $\rho_i(t) \geq \omega_{i+1}(t)$, that is, the number of openings in stage $i+1$ could easily be filled by individuals leaving stage i. Thus, we partitioned individuals leaving stage i ($\rho_i(t)$) into those moving up to the next stage,

$$\mu_i(t) = \omega_{i+1}(t) \tag{S3a}$$

and those leaving the system,

$$\lambda_i(t) = \rho_i(t) - \omega_{i+1}(t) \ . \tag{S3b}$$

However, there were two other scenarios that occasionally occurred. First, when $\omega_i(t) < 0$ (i.e., too few individuals were leaving stage i than possible, given the change in stage from year to year), we adjusted $\rho_i(t))$ as

$$\rho_i(t) = N_i(t+1) - N_i(t) \tag{S4a}$$

in order to make $\omega_i(t)$ non-negative,

$$\mu_{i-1}(t) = \omega_i(t) = 0 \ . \tag{S4b}$$

Second, when $\rho_i(t) < \omega_{i+1}(t)$ (i.e., too few individuals were leaving stage i to fill openings in stage $i+1$), we either increased the number of individuals leaving stage i when possible, or else assumed the remaining openings were filled by individuals from outside the system being modeled (e.g., coming from other scientific disciplines or returning to academia after having previously left).

Estimation Details: A to T transition

Each year within each simulation was run over time according to the following steps.

First, we estimated the number of retiring tenured professors by eqn. (S1) with $i = T$. We estimated the number of assistant professors needed to fill these tenured slots by eqn. (S2) with $i = T$. If this was a negative number of assistant professors, we adjusted it according to eqn. (S4). We estimated the number of assistant professors being tenured (and thus available to fill T slots) by eqn. (S1) with $i = A$. If $\rho_A(t) < \omega_T(t)$ (i.e., too few assistant professors were estimated as being tenured), we adjusted $\rho_A(t)$ as

$$\rho_A(t) = \omega_T(t) \tag{S5}$$

i.e., assuming that more assistant professors were tenured than initially estimated. We did not pull individuals from outside the system at this transition as it these seem likely to be rare (e.g, that an individual transitions from an assistant professor in one field to a tenured professor in a different field, or from a non-academic position to a tenured position). Otherwise, if $\rho_A(t) \geq \omega_T(t)$ we used eqn. (S3) to estimate the transition rates with $i = A$. This method effectively assumes that the rate individuals move from A to T is driven by the rate tenured professors retire (ρ_T) and that any 'excess' assistant professors receiving tenure leave the system. We refer to this as a 'demand' view of faculty turnover ('demand' in terms of empty T slots determines the A to T transition).

We thus consider a second alternative scenario, what we call a 'supply' view of faculty turnover, where 'supply' in terms of assistant professors receiving tenure determines the A to T transition. For this method, we estimated the number of retiring tenured professors by eqn. (S1) with $i = T$, estimated the number of assistant professor receiving tenure by eqn. (S1) with $i = A$, and calculated the change in the T stage as

$$\Delta_T(t) = N_T(t+1) - N_T(t) \; . \tag{S6}$$

If $\Delta_T(t) > \rho_A(t)$ (i.e., too few assistant professors were estimated as being tenured to fill the minimum number of T slots), we adjusted $\rho_A(t)$ as

$$\rho_A(t) = \Delta_T(t) \tag{S7}$$

i.e., assuming that more assistant professors were tenured than initially estimate, and set $\rho_T(t) = 0$ (no tenured professors retire this year). Otherwise, if $\Delta_T(t) < \rho_A(t)$, we set

$$\rho_T(t) = \rho_A(t) - \Delta_T(t) \; , \tag{S8}$$

i.e., that retirement of T is assumed to exactly offset the number of A being tenured, minus the new T slots that have become available.

The 'demand' scenario likely overestimates the number of faculty receiving tenure and then leaving academia, while the 'supply' scenario likely underestimates the number of faculty leaving academia after tenure and before retirement. We run simulations under both scenarios to serve as upper and lower bounds.

Estimation Details: P to A transition

Next, we estimated the number of postdoctoral researchers needed to fill these assistant professor slots by eqn. (S2) with $i = A$. We estimated the number of postdoctoral researchers available to fill A slots by eqn. (S1) with $i = P$. If $\rho_P(t) < \omega_A(t)$ (i.e., too few postdoctoral researchers were estimated as being available), we adjusted $\rho_P(t)$ as

$$\rho_P(t) = \omega_A(t) \tag{S9}$$

i.e., assuming that more postdoctoral researchers were hired than initially estimated. Otherwise, if $\rho_P(t) \geq \omega_A(t)$ we used eqn. (S3) to estimate the transition rates with $i = P$.

Estimation Details: G to P transition

Next, we estimated the number of graduate students needed to fill these postdoc slots by eqn. (S2) with $i = P$. We estimated the number of graduate students leaving that stage by eqn. (S1) with $i = G$. We assumed that only students leaving the G stage with a PhD degree can fill the P slots, so we estimated the number of graduate students available to fill P slots by $D_G(t)$, the number of PhD degrees granted in year t. If $D_G(t) \geq \omega_P(t)$ we used a modified version of eqn. (S3) to estimate the transition rates where individuals moving from stage G to stage P as

$$\mu_G(t) = \omega_P(t) \ , \tag{S10a}$$

those leaving the system with a PhD degree as

$$\lambda_G(t) = D_G(t) - \omega_P(t) \ , \tag{S10b}$$

and those leaving stage G before their degree as

$$\delta_G(t) = \rho_G(t) - D_G(t) \ . \tag{S10c}$$

Estimation Details: U to G transition

Finally, we estimated the number of undergraduate students needed to fill these graduate student slots by eqn. (S2) with $i = G$. We assumed that only students leaving the U stage with a degree can fill the G slots, so we estimated the number of undergraduate students available to fill G slots by $D_U(t)$, the number of undergraduate degrees granted in year t. We used a modified version of eqn. (S3) to estimate the transition rates where individuals moving from stage U to stage G as

$$\mu_U(t) = \omega_G(t) \tag{S11a}$$

and those leaving the system with an undergraduate degree as

$$\lambda_U(t) = D_U(t) - \omega_G(t) \ . \tag{S11b}$$

Estimation Details: Sub-partitions

Since ethnic/racial composition may vary within each stage (especially for longer career stages), we split some stages into sub-partitions. This enabled us to model different racial/ethnic compositions for each sub-partition within a stage. This also ensured that when individuals were moved out of a partitioned stage, they were taken from the oldest sub-partition. We split the graduate student stage into two sub-partitions and split the tenured professor stage into five sub-partition. We assumed that graduate students spent 3 years in the first sub-partition (approximately until qualifying exams) and then $\tau_G - 3$ in the second partition. We assumed that tenured professors spent $\tau_T/5$ in each of the five sub-partitions. Transitions between sub-partition were estimated based on turnover time. Graduate students leaving the system before receiving a degree ($\delta_G(t)$) were pulled from both sub-partitions (half from each), but graduate students leaving the stage with a doctoral degree were assumed to come only from the second sub-partition. Tenured professors retiring ($\rho_T(t)$) were pulled only from the last sub-partition.

References

[1] Shaw AK, Stanton DE. Leaks in the pipeline: separating demographic inertia from ongoing gender differences in academia. Proceedings of the Royal Society of London Series B: Biological Sciences. 2012;279(1743):3736–3741. Available from: http://rspb.royalsocietypublishing.org/content/279/1743/3736.short.
