# Supplementary material for: Differential retention contributes to racial/ethnic disparity in U.S. academia
Source: PLoS One. 2021 Dec 1;16(12):e0259710. doi: 10.1371/journal.pone.0259710 (PMC8635368; doi:10.1371/journal.pone.0259710)
Supplement: S3 File — (PDF) [file pone.0259710.s016.pdf]

Supplementary File S3 for

Differential retention contributes to racial/ethnic disparity in U.S. academia

Allison K. Shaw[1*], Chiara Accolla[1], Jeremy M. Chacón[1], Taryn L. Mueller[1], Maxime Vaugeois[1], Ya Yang[2], Nitin Sekar[3], Daniel E. Stanton[1]

[1]Department of Ecology, Evolution and Behavior, University of Minnesota-Twin Cities, Saint Paul, MN 55108
[2]Department of Plant and Microbial Biology, University of Minnesota-Twin Cities, Saint Paul, MN 55108
[3]Wildlife and Habitats Division, WWF India, New Delhi, Delhi 110003, India *To whom correspondence should be addressed; E-mail: ashaw@umn.edu.

Model simulations

Simulation details

With our model structure in place, we then simulated the flow of individuals through the system. We assumed that at each transition, the fraction of individuals staying in the system versus moving outside did not vary with race/ethnicity (i.e., individuals of different races were equally likely to stay in the system). Therefore individuals entering a given stage were drawn from the stage below in proportion to their representation in the lower stage. We calculated $n_i(t, k)$ the simulated number of individuals of each race/ethnicity k in each stage i over time t as follows. The initial number of individuals of each race/ethnicity was taken from National Science Foundation data in a starting year t_0, except for the case of postdoctoral fellows where race/ethnicity data were not available before 2010. In this case, we assumed initial proportion for each race/ethnicity that was the average of the values for graduate students and assistant professors. See Table S2 for data sources.

The survey data for undergraduate degrees and enrolled graduate students only included race/ethnicity data for US citizens and permanent residents; temporary residents were reported as a separate category with no race/ethnicity data. Temporary residents make up a large proportion of graduate students and have a different racial/ethnic composition than US citizens and permanent residents (see Figure S5). In contrast, survey data for graduate degrees did report race/ethnicity data for all graduates across residency types, so we used these data at the transition point from graduate students G to postdoctoral researchers P. Race/ethnicity data by citizenship for PhD degrees was not available

before 2000. However, data on race/ethnicity data by citizenship for the doctoral workforce was available for the years 1991 and 1993, and was thus used and interpolated to approximate race/ethnicity data for temporary resident PhD recipients between 1991 and 1999.

Next, for each year going forward, we fed in NSF data on racial/ethnic composition at a particular stage (e.g., undergraduate students), and used our model to predict the racial/ethnic composition at the other stages (e.g., graduate students). We calculated the number of individuals of race/ethnicity k in each stage in the next year $(t+1)$. The number of graduate students is given by

$$n_G(t+1,k,1) = \underbrace{n_G(t,k,1)}_{\text{initial}} - \underbrace{\beta_G(t) f_G(t,k,1)}_{\text{move up}} - \underbrace{0.5\delta_G(t) f_G(t,k,1)}_{\text{leave system}} + \underbrace{\mu_U(,t) f_U(t,k)}_{\text{move in}} \tag{S1a}$$

for the first sub-partition in G and

$$n_G(t+1,k,2) = \underbrace{n_G(t,k,2)}_{\text{initial}} - \underbrace{D_G(t) f_G(t,k,2)}_{\text{graduate}} - \underbrace{0.5\delta_G(t) f_G(t,k,2)}_{\text{leave system}} + \underbrace{\beta_G(t) f_G(t,k,1)}_{\text{move in}} \tag{S1b}$$

for the second sub-partition in G, where $f_i(t,k)$ is the fraction of individuals of race/ethnicity k in stage i in year t and $\beta_G(t)$ is the number of individuals that move between G sub-partitions in year t.

The number of postdoctoral researchers is given by

$$n_P(t+1,k) = \underbrace{n_P(t,k)}_{\text{initial}} - \underbrace{\mu_P(t) f_P(t,k)}_{\text{move up}} - \underbrace{\lambda_P(t) f_P(t,k)}_{\text{leave system}} + \underbrace{\mu_G(t) R(t) f_G(t,k,2)}_{\text{move in (perm. res.)}} + \underbrace{\mu_G(t)(1-R(t)) V(t,k)}_{\text{move in (temp. res.)}} \,. \tag{S1c}$$

where $R(t)$ is the fraction of PhD degrees that go to U.S. citizens and permanent residents in year t (thus, $1-R(t)$ go to temporary residents), and $V(t,k)$ is the fraction of U.S. temporary resident PhD recipients of race/ethnicity k in year t. The number of assistant professors is given by

$$n_A(t+1,k) = \underbrace{n_A(t,k)}_{\text{initial}} - \underbrace{\mu_A(t) f_A(t,k)}_{\text{move up}} - \underbrace{\lambda_A(t) f_A(t,k)}_{\text{leave system}} + \underbrace{\mu_P(t) f_P(t,k)}_{\text{move in}} \,. \tag{S1d}$$

The number of tenured professors is given by

$$n_T(t+1,k,1) = \underbrace{n_T(t,k,1)}_{\text{initial}} - \underbrace{\beta_T(t,1) f_T(t,k,1)}_{\text{move up}} + \underbrace{\mu_A(t) f_A(t,k)}_{\text{move in}} \tag{S1e}$$

for the first sub-partition in T,

$$n_T(t+1,k,j) = \underbrace{n_T(t,k,j)}_{\text{initial}} - \underbrace{\beta_T(t,j) f_T(t,k,j)}_{\text{move up}} + \underbrace{\beta_T(t,j-1) f_T(t,k,j-1)}_{\text{move in}} \tag{S1f}$$

for sub-partitions 2 through 4 ($j = 2, 3, 4$) in T, and

$$n_T(t+1,k,5) = \underbrace{n_T(t,k,5)}_{\text{initial}} - \underbrace{\rho_T(t) f_T(t,k,5)}_{\text{retire}} + \underbrace{\beta_T(t,4) f_T(t,k,4)}_{\text{move in}} \tag{S1g}$$

for the last (fifth) partition in T.

Simulation scenarios

We considered four types of scenarios for our simulations (based on turnover rate and turnover type), which capture uncertainty in the details surrounding transitions for faculty in academia. Although we found NSF data on the average length of time spent as a PhD student and as a postdoctoral researcher (Table S1), we could not find similar data on the average time spent on the tenure-track or as a tenured professor. Instead, we considered (i) a 'slow' turnover within the faculty, estimating the time spent on the tenure-track (τ_A) as 8 years and the time spent as a tenured professor (τ_T) as 30 years, and (ii) a 'fast' turnover within the faculty, estimating τ_A as 5 years and τ_T as 20 years. We also considered that the rate individuals moved between the A and T stages was driven by (i) 'supply' (i.e, rate of A achieving tenure), and (ii) 'demand' (i.e., rate of T retiring). We thus considered four combinations of scenarios: fast-supply, fast-demand, slow-supply and slow-demand.

Simulation sets

We ran three sets of simulations, each run under the four scenarios described above.

First, to study the overall effects of retention (Figure 2 in the paper), we started simulations in year $t_0 = 1991$ and ran them for 25 years (the full range of available data), feeding NSF data on the race/ethnicity of graduating undergraduates, and simulating the expected race/ethnicity of graduate students, postdoctoral researchers, assistant professors, and tenured professors. We used five initial groups for race/ethnicity: 'White', 'Asian or Pacific Islander', 'Black', 'Hispanic', and 'Native American/Alaskan Native'. Around 2010 (year differs slightly across academic stages), the group 'Asian or Pacific Islander' was split into 'Asian' and 'Native Hawaiian or Other Pacific Islander' in the NSF data and the group 'More than one race' was added. Accordingly, we adjusted the simulated individuals in our model starting in the year 2012 (the first year that these two new groups were available for all academic stages). We partitioned the simulated individuals in the 'Asian or Pacific Islander' group into 'Asian' and 'Native Hawaiian or Other Pacific Islander' groups based on the relative proportion of these two groups in the NSF data for 2012. Similarly, we set the proportion of simulated individuals in the 'More than one race' group based on the relative proportion of that group in the NSF 2012 data, and pulled these simulated individuals evenly from the other simulated groups.

Second, to isolate the effects of retention within each stage of academia (Figure 4 in the paper), we fed in NSF data on the race/ethnicity at each stage and quantified the expected outcome at the following stage. Specifically, we simulated expected results for graduate students based on our model run with NSF undergraduate student data, expected results for postdoctoral researchers and assistant professors based on NSF graduate student data, and expected results for tenured professors based on NSF assistant professor data. This second set of simulations was also started in the year $t_0 = 1991$, running them for 25 years.

Third, to examine how the effect of specific transitions within academia changed over time, we started simulations in different starting years ($t_0 = 1991, 1996, 2001, 2006$) and ran each simulation for 10 years. Here again we simulated expected results for each stage based on our model run with NSF data at the previous stage.

All simulations and calculations were done using *Matlab*.

Testing model predictions

Finally, we compared the racial/ethnic composition predicted by our null model to the actual composition from NSF data. We quantified this comparison with the metric

$$\theta = \frac{d_i(t,k) - f_i(t,k)}{f_i(t,k)} \tag{S2}$$

where $d_i(t,k)$ and $f_i(t,k)$ are the NSF data and model prediction, respectively, of the proportion of stage i in year t that is made up of race/ethnicity k. Here, $\theta > 0$ indicates that a racial/ethnic group has higher representation in a stage than is predicted by the null model and $\theta < 0$ means lower representation than predicted.

We calculated confidence intervals around θ values, as follows. For each combination of $d_i(t,k)$ and $f_i(t,k)$, we considered what effect an error of $\epsilon = 5\%$ would have. We calculated four bounds to the θ metric:

$$\theta_1' = \frac{(1-\epsilon)d_i(t,k) - (1-\epsilon)(t,k)}{(1-\epsilon)f_i(t,k)} \tag{S3a}$$

$$\theta_2' = \frac{(1-\epsilon)d_i(t,k) - (1+\epsilon)(t,k)}{(1+\epsilon)f_i(t,k)} \tag{S3b}$$

$$\theta_3' = \frac{(1+\epsilon)d_i(t,k) - (1-\epsilon)(t,k)}{(1-\epsilon)f_i(t,k)} \tag{S3c}$$

$$\theta_4' = \frac{(1+\epsilon)d_i(t,k) - (1+\epsilon)(t,k)}{(1+\epsilon)f_i(t,k)} \tag{S3d}$$

and used the largest and smallest value of these four to set the upper and lower bounds of the confidence interval around the θ value.

Supplementary Results

In addition to the results in the main text, several supplementary results are included below. Table S4 provides numerical value of representation in each stage for each race/ethnicity.

Figure S7 shows a comparison of representation comparing the model results, academia data and census data. Figure S8 shows the temporal trends in the θ metric value. Figure S9 shows a comparison of two model versions – one accounting for the race/ethnicity of temporary resident international scholars who receive their PhDs in the U.S., and one ignoring the race/ethnicity of this group.
