# Supplementary material for: Differential retention contributes to racial/ethnic disparity in U.S. academia
Source: PLoS One. 2021 Dec 1;16(12):e0259710. doi: 10.1371/journal.pone.0259710 (PMC8635368; doi:10.1371/journal.pone.0259710)
Supplement: S4 File — (PDF) [file pone.0259710.s017.pdf]

Supplementary File S4 for

Differential retention contributes to racial/ethnic disparity in U.S. academia

Allison K. Shaw[1*], Chiara Accolla[1], Jeremy M. Chacón[1], Taryn L. Mueller[1], Maxime Vaugeois[1], Ya Yang[2], Nitin Sekar[3], Daniel E. Stanton[1]

[1]Department of Ecology, Evolution and Behavior, University of Minnesota-Twin Cities, Saint Paul, MN 55108
[2]Department of Plant and Microbial Biology, University of Minnesota-Twin Cities, Saint Paul, MN 55108
[3]Wildlife and Habitats Division, WWF India, New Delhi, Delhi 110003, India *To whom correspondence should be addressed; E-mail: ashaw@umn.edu.

Data Report Details

Below are details of each data source used.
[08-307] NSF Publication 08-307. 2008 National Science Foundation, Division of Science Resources Statistics, Postdoc Participation of Science, Engineering, and Health Doctorate Recipients. (http://www.nsf.gov/statistics/infbrief/nsf08307)
2008 report, Table 2: Median duration of most recently completed postdoc

[GSPD] Survey of Graduate Students and Postdoctorates in Science and Engineering. (https://www.nsf.gov/statistics/srvygradpostdoc/)
2018 report, Table 1-9a: number of graduate students by science field for 1975–2018
2018 report, Table 1-10a: number of graduatd students by engineering field for 1975–2018
2018 report, Table 1-9b: number of postdoctoral researchers by science field for 1975–2018
2018 report, Table 1-10b: number of postdoctoral researchers by engineering field for 1975–2018
2010 report, Table 34: postdoctoral researchers, by race/ethnicity for 2010
2016 report, Table 34: postdoctoral researchers, by race/ethnicity for 2011-2016
2017 report, Table 2-2: postdoctoral researchers, by race/ethnicity for 2017
2018 report, Table 2-2: postdoctoral researchers, by race/ethnicity for 2018

[S&E Degrees] Science and Engineering Degrees: 1966–2012. (https://www.nsf.gov/statistics/2015/nsf15326/)
2015 report, Table 5: number of bachelor's degrees by field for 1966-2012
2015 report, Table 19: number of PhD degrees by field for 1966-2012

[SED] Survey of Earned Doctorates.
(https://www.nsf.gov/statistics/srvydoctorates/)
2014 report, Table 17: doctorate recipients, by broad field of study and citizenship for 1984-2014 (every 5 years)
2015 report, Table 17: doctorate recipients, by broad field of study and citizenship for 1985-2015 (every 5 years)
2016 report, Table 17: doctorate recipients, by broad field of study and citizenship for 1986-2016 (every 5 years)
2017 report, Table 17: doctorate recipients, by broad field of study and citizenship for 1987-2017 (every 5 years)
2018 report, Table 17: doctorate recipients, by broad field of study and citizenship for 1988-2018 (every 5 years)
2010 report, Table 19: doctorate recipients, by race/ethnicity and citizenship for 2000–2010
2018 report, Table 19: doctorate recipients, by race/ethnicity and citizenship for 2009–2018

[SE-ind] Science and Engineering Indicators, National Science Board.
(https://ncses.nsf.gov/indicators)
2019 report, Table S3-7: number of assistant and tenured professors by field for 1973-2017
2018 report, Table 2-3: median time to degree by field for 1985-2015

[WMPD] Women, Minorities, and Persons with Disabilities in Science and Engineering report.
(https://www.nsf.gov/statistics/women/)
2019 report, Table 5-3: number of bachelor's degrees by field for 2006-2016
2019 report, Table 7-4: number of PhD degrees by field for 2006–2016
1994 report, Table 5-19: bachelors degrees by race/ethnicity for 1981-1991
2002 report, Table 3-8: bachelors degrees by race/ethnicity for 1990-1998
2009 report, Table C6: bachelors degrees by race/ethnicity for 1996-2007
2019 report, Table 5-3: bachelors degrees by race/ethnicity for 2006-2016
2002 report, Table 4-6: graduate students by race/ethnicity for 1990-1999
2009 report, Table D-1: graduate students by race/ethnicity for 1999-2006
2011 report, Table 3-1: graduate students by race/ethnicity for 2008-2010
2013 report, Table 3-1: graduate students by race/ethnicity for 2012
2017 report, Table 3-1: graduate students by race/ethnicity for 2014
2019 report, Table 3-1: graduate students by race/ethnicity for 2016
1994 report, Table 8-11: PhD workforce by race/ethnicity and citizenship for 1991
1996 report, Table 5-33: PhD workforce by race/ethnicity and citizenship for 1993
1994 report, Table 8-18: faculty by race/ethnicity for 1991
1996 report, Table 5-28: faculty by race/ethnicity for 1993
1998 report, Table 5-10: faculty by race/ethnicity for 1995
2000 report, Table 5-19: faculty by race/ethnicity for 1997
2004 report, Table H-26: faculty by race/ethnicity for 2001
2007 report, Table H-28: faculty by race/ethnicity for 2003
2009 report, Table H-28: faculty by race/ethnicity for 2006
2011 report, Table 9-26: faculty by race/ethnicity for 2008

2013 report, Table 9-26: faculty by race/ethnicity for 2010
2015 report, Table 9-26: faculty by race/ethnicity for 2013
2017 report, Table 9-26: faculty by race/ethnicity for 2015
2019 report, Table 9-26: faculty by race/ethnicity for 2017
